# Genome-Wide Patterns of Codon Bias Are Shaped by Natural Selection in the Purple Sea Urchin, *Strongylocentrotus purpuratus*

**DOI:** 10.1534/g3.113.005769

**Published:** 2013-07-01

**Authors:** Kord M. Kober, Grant H. Pogson

**Affiliations:** * Department of Ecology and Evolutionary Biology, University of California, Santa Cruz, California 95064; † Department of Physiological Nursing, University of California, San Francisco, California 94143

**Keywords:** codon bias, translational selection, sea urchin, antagonistic pleiotropy, mRNA stability, mutational bias

## Abstract

Codon usage bias has been documented in a wide diversity of species, but the relative contributions of mutational bias and various forms of natural selection remain unclear. Here, we describe for the first time genome-wide patterns of codon bias at 4623 genes in the purple sea urchin, *Strongylocentrotus purpuratus*. Preferred codons were identified at 18 amino acids that exclusively used G or C at third positions, which contrasted with the strong AT bias of the genome (overall GC content is 36.9%). The GC content of third positions and coding regions exhibited significant correlations with the magnitude of codon bias. In contrast, the GC content of introns and flanking regions was indistinguishable from the genome-wide background, which suggested a limited contribution of mutational bias to synonymous codon usage. Five distinct clusters of genes were identified that had significantly different synonymous codon usage patterns. A significant correlation was observed between codon bias and mRNA expression supporting translational selection, but this relationship was driven by only one highly biased cluster that represented only 8.6% of all genes. In all five clusters preferred codons were evolutionarily conserved to a similar degree despite differences in their synonymous codon usage distributions and magnitude of codon bias. The third positions of preferred codons in two codon usage groups also paired significantly more often in stems than in loops of mRNA secondary structure predictions, which suggested that codon bias might also affect mRNA stability. Our results suggest that mutational bias has played a minor role in determining codon bias in *S. purpuratus* and that preferred codon usage may be heterogeneous across different genes and subject to different forms of natural selection.

The nonrandom usage of synonymous codons (codon bias) has been observed across all domains of life and varies in magnitude among closely related species and among genes within a genome (Grantham *et al.* 1980). The identity of the most frequently used synonymous codons within a genome may be similar or variable among loci. This cause of this variation has been attributed to mutational bias, random genetic drift, and natural selection, but the relative contributions of each remain unclear (Sharp and Li 1986; Bulmer 1991). Because synonymous codon substitutions do not change protein sequences, it has long been suggested that such changes are neutral and thus reflect of nonadaptive processes (King and Jukes 1969). Indeed, in broad taxonomic studies examining genome-wide patterns of codon bias researchers generally have found mutation processes may play a major role across broad domains of life (Chen *et al.* 2004). However, the variation available in the degeneracy of the genetic code and the multistep nature of transcription through translation may provide subtle opportunities on which natural selection may act (Clarke 1970; Sharp *et al.* 1995), and many researchers have detected subtle forces of selection at play (reviewed by Duret 2002; Hershberg and Petrov 2008; Plotkin and Kudla 2011).

The possibility that natural selection could affect the rate of polypeptide synthesis through the matching of transfer RNA abundance and codon usage is not new (Zuckerkandl and Pauling 1965). The *in vivo* rate and accuracy of translation are affected by the use or disuse of “major” codons in *Escherichia coli* (Tuller *et al.* 2010; Navon and Pilpel 2011), and early observations of genome-wide synonymous codon usage in *D. melanogaster* and *C. elegans* showed a bias toward a subset of codons (Shields *et al.* 1988; Stenico *et al.* 1994). This set of preferred codons corresponds to the most abundant tRNAs in the cell and, by proxy, the number of tRNA gene copies in the genome (Moriyama and Powell 1997; Duret 2000). Subtle effects on fitness may be realized by a decrease in the cost of protein synthesis (Sharp *et al.* 1995) or by an increase in growth rate (Andersson and Kurland 1990; Kudla *et al.* 2009). Accordingly, highly transcribed genes have been found to be the most biased loci in both *Drosophila* and yeast (Duret and Mouchiroud 1999). Furthermore, in *Drosophila* the magnitude of codon bias is accentuated at genes expressed during the high growth rate larval stage (Powell *et al.* 1993; Vicario *et al.* 2008).

Early studies on the nature of the synonymous codon usage in yeast identified two relatively homogeneous groups of genes with different patterns of synonymous codon preference (Sharp *et al.* 1986). These different patterns proved to be biologically significant because they differentiated highly and lowly expressed genes. The selective advantages of translational efficiency and accuracy are clear and might explain the greater codon bias of highly expressed genes sharing one pattern of codon usage. However, it is unclear what selective processes, if any, might influence the persistence of the translationally suboptimal synonymous codons of the other group. It is possible that selection might influence the frequency of both major and minor codons in some taxa (Kreitman and Antezana 2000). This antagonistic pleiotropy hypothesis posits that minor codon frequencies may be driven by conflicting adaptive forces acting in competition on synonymous codons (Akashi and Eyre-Walker 1998). According to this hypothesis, major codons reflect translationally optimal codons, whereas minor codons represent translationally suboptimal codons. Translationally suboptimal codons, however, are still subject to other forms of selection. Different forms of natural selection on synonymous codons may thus compete for the synonymous state of the same codon.

Natural selection and mutation affect base composition and DNA sequence variation at the level of the genome, the gene, and at individual sites within genes. Genome-wide studies are capable of identifying major effectors of codon bias but the focus on major and minor codons may miss more subtle forms of natural selection. Identifying patterns of synonymous codon usage has proven useful in identifying group of genes likely under translational selection (Sharp *et al.* 1986). However, more than two clusters of synonymous codon usage patterns have been recently identified in both *E. coli* and *Bacillus subtilus*, and only one cluster in each species was associated with translational selection (Bailly-Bechet *et al.* 2006). These results are intriguing because they suggest that different patterns of synonymous codon usage might exist among different groups of genes and that multiple forms of selection (or mutation) may be acting on the different groups. Before this study, it is unclear whether multiple clusters of codon usage groups occur outside of bacteria and yeast.

Previous work on codon bias in metazoans has been performed primarily in the protostomes such as *Drosophila* and *C. elegans*. In deuterostomes, codon bias has been studied in vertebrates such as *Xenopus* (Musto *et al.* 2001) and *Gallus* (Rao *et al.* 2011), as well as in mammals (Chamary *et al.* 2006). However, there are a large number of nonchordate deuterostome species with large effective population sizes that could enable natural selection to act effectively on the subtle selection coefficients associated with synonymous codon usage (*i.e.*, *N_e_s* ≈ 1). Here, we investigate the patterns of synonymous codon usage in the purple sea urchin, *Strongylocentrotus purpuratus*. The purple sea urchin is an excellent candidate nonvertebrate deuterostome species in which to study codon bias. It is a widely distributed, broadcast spawner with a free swimming larval period of 2–3 months (Strathmann 1978) that results in a large effective population size, extensive levels of nucleotide polymorphism, minimal population structuring, and a well-supported phylogeny (Britten *et al.* 1978; Palumbi and Wilson 1990; Addison and Pogson 2009; Kober and Bernardi 2013). *S. purpuratus* has been studied as a model organism for early development for more than a century and a half (Pederson 2006), has a well-annotated genome assembly and transcriptome (Sodergren *et al.* 2006; Cameron *et al.* 2009), and is phylogenetically positioned to provide insight into vertebrate model systems (Cameron and Davidson 2007). Furthermore, the majority of its predicted ~23,300 genes reside in regions with a narrow range of GC between 35 and 39% (Sodergren *et al.* 2006), thus minimizing the contribution of variable mutational bias across the genome to patterns of codon bias.

The objectives of the present study were to (1) investigate the presence of codon bias and the identity of preferred codons in the *S. purpuratus* genome, (2) assess the presence of distinct clusters of codon usage, and (3) examine the relative contributions of translational selection and selection on mRNA stability on the observed patterns of codon bias. We describe for the first time the existence of codon bias a marine invertebrate and show how mutational bias is an unlikely explanation for the nonrandom usage of synonymous codons. We document the existence of five distinct clusters of genes with different codon usage patterns and show how translational selection and selection for mRNA secondary structure differentially affect subsets of the codon usage groups. Finally, we identify the presence of five codon usage clusters in *Drosophila melanogaster* and show how translational selection is acting upon one group (as in *S. purpuratus*), suggesting the wide generality of these patterns.

## Materials and Methods

### *S. purpuratus* genomics

*S. purpuratus* genomic sequences were obtained from the Baylor College of Medicine (http://www.hgsc.bcm.tmc.edu/ftp-archive/Spurpuratus/fasta/Spur_v2.1/). The gene models (*i.e.*, genomic coordinates) of the Official Gene Set (OGS), GLEAN3_b-2.1-revised.gff and coding sequence annotations were obtained from the Sea Urchin Genome Database SpBase.org (Cameron *et al.* 2009). Coding sequence was obtained from the genomic sequence using the OGS gene models. Estimates of mRNA expression levels for early developmental stages (2, 15, 30, 48, and 72 hr) for all *S. purpuratus* genes were downloaded from the National Institute of Dental and Craniofacial Research (http://urchin.nidcr.nih.gov/blast/exp.html). Following Wei *et al.* (2006), we defined high expression as having an AU >25,000 (Arbitrary Units) and used the maximum expression observed across all five stages for all analyses.

Although the genome assembly is a high-quality draft and the OGS a well-annotated set of gene models, we applied a series of filters to minimize errors in our gene models. Each manually annotated gene prediction in the OGS underwent basic validation using CodonW (http://codonw.sourceforge.net/). Sequences identified by CodonW to have nonrecognized start codons, nontranslatable codons, partial last codons, and internal stop codons were discarded. Gene predictions without annotation data in SpBase (or annotated as “duplicates”), or as having excessive repeats or lacking sufficient information (*i.e.*, having insufficient degrees of freedom) as identified by SCUMBLE (Kloster and Tang 2008) also were excluded. Of the remaining gene models, those with maximum expression scores less than 200 AU were removed. Finally, genes with identical expression scores for all time periods were treated as unhandled duplicates and those with the smallest codon bias scores (Wright’s Nc; see the section *Genome-wide patterns of codon bias*) were retained. These filters left 4623 unique gene models for the study. Gene ontology (GO) terms (Harris *et al.* 2004) for 4232 of the 4623 genes were annotated using blastx interrogation of the SWISSPROT database (v. b2g.may10) with Blast2GO v. 2.4.9 (Conesa *et al.* 2005). Fisher’s exact test with false discovery rate multiple testing corrections were used to assess enrichment of GO terms associated with genes in each group relative to entire set.

Secondarily filtered counts of tRNA genes predicted by tRNAscan- SE (Lowe and Eddy 1997) were obtained from the UCSC tRNA lab as a conservative set (http://gtrnadb.ucsc.edu/Spurp/Spurp-stats.html). Amino acids identified by a single tRNA gene were excluded from the analysis. tRNA gene species counts were used as proxies for tRNA gene concentrations. General wobble rules were used to identify the synonymous codons recognized by isoaccepting tRNA genes. Following Ikemura (1985), the frequency of use for tRNA genes with anticodons recognizing a single codon was estimated from the observed frequency of synonymous codon usage. For tRNA genes with anticodons identifying multiple codons, their frequency of use was the sum of the observed frequencies of each synonymous codon (Ikemura 1985). The frequencies of tRNA gene copy numbers and synonymous codons for each tRNA species were normalized to 1.0 using the greatest frequency observed. We tested for relationships between frequencies of the isoaccepting tRNA gene copy numbers and the usage of their corresponding synonymous codons. Correlations were determined using the cor() function in the R statistical package (Ihaka and Gentleman 1996) for the entire dataset and separately for each codon usage group (see the section *Clustering genes by codon usage distributions* below).

Data manipulations were performed using the Jim Kent’s source tools (http://hgwdev.cse.ucsc.edu/~kent/src), the UCSC Sea Urchin Genome Browser strPur2 (Karolchik *et al.* 2007), BioPerl (Stajich *et al.* 2002), and BioPython (Cock *et al.* 2009). Comparative alignments and *dS* estimates for 2954 genes *Allocentrotus fragilis* and *S. franciscanus* genes were kindly provided by David Garfield (Oliver *et al.* 2010).

### Codon bias measures

We used the effective number of codons, Nc (Wright 1990), to estimate codon bias. Nc (or ENC) is analogous to the effective number of alleles at a locus and ranges from 61 (random usage) to 20 (one codon per amino acid). To account for background nucleotide composition, Nc′ scores also were determined using ENCprime (Novembre 2002). Background composition was determined from the genomic nucleotide sequences of all nucleotides 1000 bp upstream and downstream of transcript initiation start and stop sites, respectively. Coding sequence GC levels (GC_cds_) and third position codon GC levels (GC3) were calculated using CodonW. The GC content of flanking regions (GC_f_) was estimated from nucleotide sequences 1000 bp upstream and downstream of the predicted start and stop codons, respectively. GC levels for introns (GC_i_) were calculated for all genes possessing introns (n = 4389). These data for S. *purpuratus* are provided in File S1.

To examine the relative contributions of individual amino acids to the overall codon bias of a gene, sENC-X scores were calculated for each amino acid (where X is a particular amino acid) (Moriyama and Powell 1997). Because unscaled ENC-X scores depend on the degeneracy of the amino acid (*e.g.*, fourfold degenerate amino acids have ENC-X scores between 0 and 4), the sENC-X values are reset to fall between 0 (no bias) and 1 (maximum bias) for all amino acids. ENC-X scores were estimated using the ‘cusage’ program kindly provided by Etsuko Moriyama.

To identify genome-wide preferred codons, we determined frequency of each codon’s usage within its codon family for each gene and tested the Spearman correlation between these frequencies with the overall codon bias of that gene (Nc′). The preferred codon for each amino acid was identified as that with the most significant negative correlation with Nc′ (Hershberg and Petrov 2008). In other words, the genome-wide preferred codons are defined as the codons that are used more frequently as the bias of the gene increases (Supporting Information, Figure S1). The significance threshold was defined by a *P*-value smaller than 0.05/*n*, where *n* is the number of codons in the codon family. This adjustment is to correct for performing more comparisons on more degenerate codon families (Hershberg and Petrov 2008).

### Clustering genes by codon usage distributions

We used the clustering method of Bailly-Bechet *et al.* (2006) to identify groups on the basis of their codon usage patterns. Each group was defined by a distribution of synonymous codon usage that differed from group to group. The number of clusters, *S*, was optimized in terms of the information content. *S* was determined by maximizing the difference in self-consistency, $b_{g}^{\left(s\right)}$, between the real data and a null model. $b_{g}^{\left(s\right)}$ provides a value reflecting the quality of the assignment of the gene *g* to cluster *s*, where the quality of the assignment relates to how close the value is to unity (Bailly-Bechet *et al.* 2006). The geometric average, *B*(*S*), of all clusters provides the quality of corresponding assignments. A null distribution was generated using an artificial dataset generated from the real data and the quality of this set, *B_random_*(*S*), also was determined. The maximum difference Δ(*S*) = *B*(*S*) − *B_random_*(*S*) gives the number of clusters *S* retained. Clustering and assessments were performed using the software implementation of Bailly-Bechet *et al.* (2006). For visualization in heatmaps, codon usage frequencies were centered on a random expectation using the degeneracy of the codon. The centered value, $v_{a}^{\left(s\right)}\left(c\right)$, was calculated from the observed frequency $p_{a}^{\left(s\right)}\left(c\right)$ of group *g* for codon *c* for amino acid *a* and the expected equal usage frequency based on the degeneracy *d* of the codon:

$$v_{a}^{\left(s\right)}\left(c\right)=\frac{p_{a}^{\left(s\right)}\left(c\right)-\left(\frac{1}{d_{a}\left(c\right)}\right)}{\left(1-\frac{1}{d_{a}\left(c\right)}\right)}$$ (1)

We also applied the probabilistic model of codon bias implemented by SCUMBLE v 1.0 (Kloster and Tang 2008) to identify clusters of genes with similar patterns of codon usage. The algorithm fits a codon usage model in which each gene *g* is assigned a given number of “offsets” *β_i_*(*g*). An offset indicates to what extent gene *g* is affected by estimated bias (‘trend’) number *i*. A “preference function” *E_i_*(*c*) for each trend indicates how much trend *i* favors/disfavors codon *c* (Kloster and Tang 2008). We explored models with 0 to 10 trends using SCUMBLE. The number of trends used for the analyses was based on the normalized variance *NV(g)* of the genes.

### Conservation of preferred synonymous codons

To test for the conservation of preferred codons, we used Akashi’s test (Akashi 1994). For all genes, each codon was identified as preferred or nonpreferred and as conserved (synonymous) or nonconserved (nonsynonymous) between *S. purpuratus* and either *A. fragilis* or *S. franciscanus* (Stoletzki and Eyre-Walker 2007) using alignments provided by David Garfield (Oliver *et al.* 2010). Preferred codons within groups delineated by the clustering methods described above were identified as having the highest frequency of usage within each separate group. Only genes with sufficient data for the contingency tables were used (n = 2349). The Woolf test for homogeneity across genes in each cluster (Woolf 1955) was implemented using woolf_test from the vcd library in the R statistical package (Ihaka and Gentleman 1996). All tables for genes clustering into different groups were combined for analysis using the Cochran–Mantel–Haenszel procedure (Cochran 1954; Mantel and Haenszel 1959) and tested using mantelhaen.test from the stats library in R. This allowed us to determine if site conservation (*i.e.*, synonymous substitutions) was independent of codon preference in any given codon usage cluster. To assess the strength of the association between conservation and codon preference, we followed Drummond and Wilke (2008) and generated a reference distribution of the odds ratio for each codon usage group by performing Akashi’s test and computing the odds ratio for 1000 randomly generated sets of synonymous codons from the genes of that group.

### Preference for mRNA folding stabilizing codons

To test if the third nucleotide positions of preferred codons were more likely to be stem-paired in RNA secondary structure, the data were stratified by gene. RNA secondary structure predictions were performed using RNAfold from the Vienna RNA Package v1.8.5 (Hofacker 2004). Nucleotides in the third position of each codon (N3) were identified as either preferred or non-preferred and as falling in a stem or loop in the folding prediction. Preferred codons for each grouping were determined as described above for the Akashi (1994) test. Then, 2 × 2 contingency tables were constructed for each gene (*e.g.*, Table S1) and Woolf tests for homogeneity and association tests using the Cochran–Mantel–Haenszel procedure were performed as described previously. This allowed us to test if folding role (*i.e.*, paired in a stem or unpaired in a loop) is independent of codon preference.

### *D. melanogaster* genomics

The genomic assembly for *D. melanogaster* was obtained from the UCSC Genome Browser (dm3, Apr. 2006, BDGP R5). A total of 11,776 gene models were obtained for the Release 5.4 Annotation Update from flyBase.org (Flybase 2007_03 Update). Each gene prediction underwent the same validation procedure described earlier for *S. purpuratus*, leaving 11,223 gene models for analyses. The coding sequence for *Notch* and 1000 bp of flanking sequence was obtained from flyBase.org. Estimates of mRNA expression were obtained from the LS mean of overall expression of *D. melanogaster (y*;*cnbwsp)* from the Affymetrix Gene Chip Drosophila Tiling 1.0R Arrays (Graze *et al.* 2009). Expression values for *Notch* were not available in these data. These gene data for *D. melanogaster* are provided in File S2.

## Results

### Genome-wide patterns of codon bias

**Figure 1:**
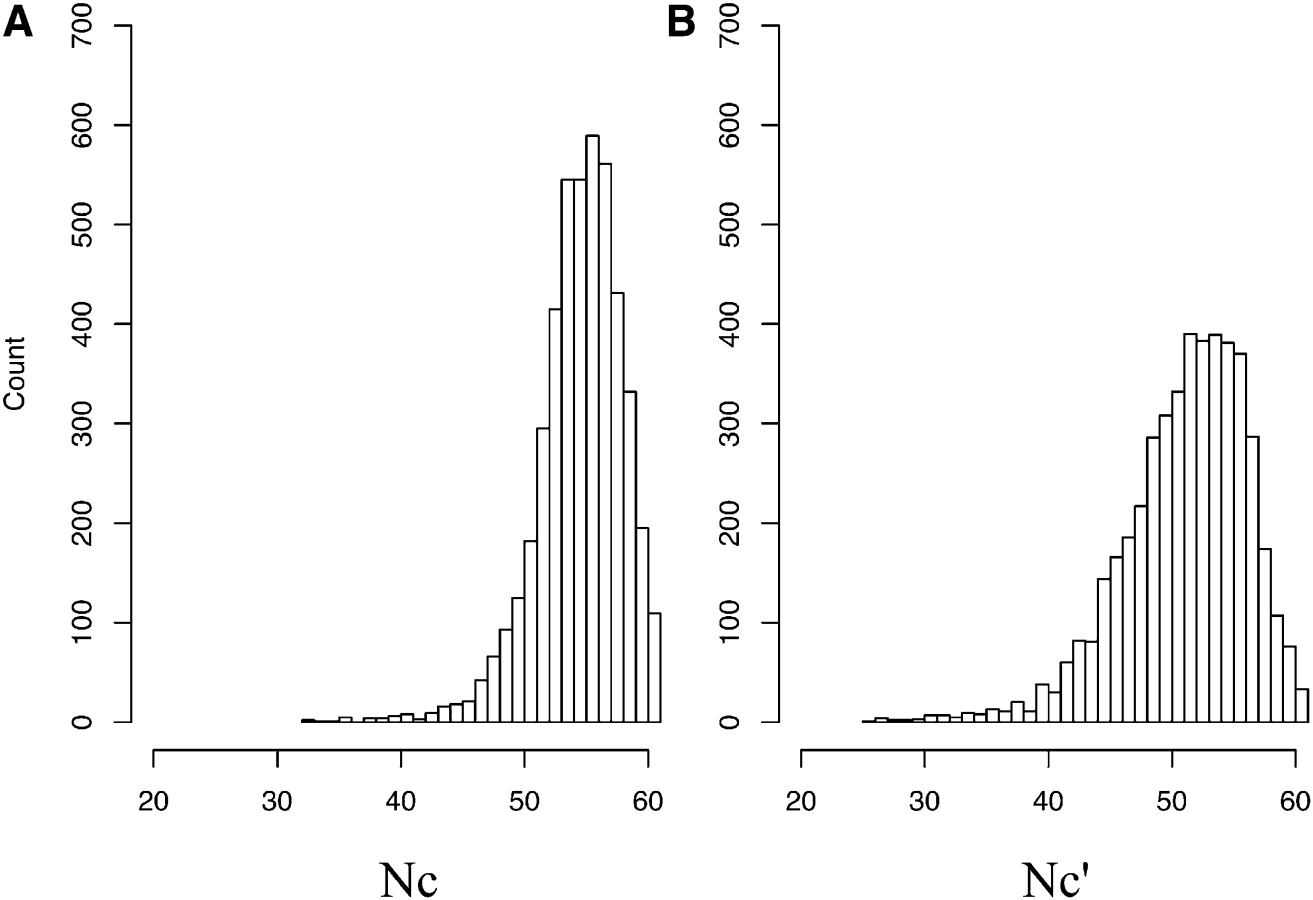
Distribution of codon bias scores of (A) Nc and (B) Nc′ in *S. purpuratus*.

The mean GC content of the *S. purpuratus* genome is 36.9% and the vast majority of the ~23,300 genes fall in regions of GC between 35 and 39% Sodergren *et al.* (2006). For the 4623 genes analyzed in the present study, the GC content of coding regions was greater than the genome-wide mean (48.4%) but the GC content of introns and flanking regions were not (35.1% and 36.3%, respectively). Figure 1 presents the overall extent of codon bias in our data. Wright’s (1990) effective number of codons (Nc) ranged from 32 to 61, exhibiting a mean of 54.5 (Figure 1). Correcting for background GC yielded Nc′ scores ranging from 25.4 to 61 with mean of 51.0.

Despite the weak overall signal of codon bias, we successfully identified preferred codons at all 18 amino acids having two or more synonymous codons (Table 1). Figure S1 shows for isoleucine (*i.e.*, Ile) how the usage of the preferred codon (ATC) increases as the magnitude of codon bias increases. In contrast to the AT bias in the *S. purpuratus* genome, all preferred codons used a C or a G at the third position and the former were used far more frequently than the latter (15 *vs.* 3; Table S2). We also observed significant genome-wide correlations between isoaccepting tRNA gene copy numbers and the usage of both synonymous codon and amino acids (Table 2). Across all groupings, synonymous codons identified by tRNA genes with a greater species counts were used more commonly. Many of the isoaccepting tRNA gene predictions were redundant for the synonymous codons they identified (not shown).

**Table 1:** Genome-wide preferred synonymous codon usage in *S. purpuratus*

	Second Base*^a^*											
	U			C			A			G		
First Base	Codon	ρ*^b^*	*P*-Value	Codon	ρ	*P*-Value	Codon	ρ	*P*-Value	Codon	ρ	*P*-Value
U	UUU (F)	0.381	7.54E-160	UCU (S)	0.191	3.94E-39	UAU (Y)	0.387	9.07E-165	**UGU (C)**	**-0.191**	**2.13E-39**
	**UUC (F)**	**-0.382**	**1.43E-160**	**UCC (S)**	**-0.279**	**2.19E-83**	**UAC (Y)**	**-0.381**	**1.12E-159**	UGC (C)	0.208	2.18E-46
	UUA (L)	0.453	2.16E-232	UCA (S)	0.231	5.12E-57	UAA (X)			UGA (X)		
	UUG (L)	0.274	1.67E-80	UCG (S)	−0.022	1.27E-01	UAG (X)			UGG (W)		
C	CUU (L)	0.208	2.28E-46	CCU (P)	0.157	7.61E-27	CAU (H)	0.242	2.24E-62	CGU (R)	−0.021	1.46E-01
	**CUC (L)**	**-0.461**	**2.87E-242**	CCA (P)	0.185	5.08E-37	**CAC (H)**	**-0.228**	**1.10E-55**	**CGC (R)**	**-0.213**	**8.89E-49**
	CUA (L)	0.253	1.65E-68	**CCC (P)**	**-0.317**	**1.19E-108**	CAA (Q)	0.382	1.73E-160	CGA (R)	0.152	3.05E-25
	CUG (L)	−0.390	2.64E-167	CCG (P)	−0.011	4.64E-01	**CAG (Q)**	**-0.378**	**2.78E-157**	CGG (R)	0.062	2.56E-05
A	AUU (I)	0.342	3.55E-127	ACU (T)	0.277	3.53E-82	AAU (N)	0.454	2.42E-233	AGU (S)	0.204	1.68E-44
	**AUC (I)**	**-0.536**	**1.00E-223**	**ACC (T)**	**-0.420**	**6.96E-197**	AAC (N)	**-0.452**	**5.70E-232**	AGC (S)	−0.230	1.61E-56
	AUA (I)	0.385	6.16E-163	ACA (T)	0.284	2.66E-86	AAA (K)	0.466	2.95E-248	AGA (R)	0.302	4.55E-98
	AUG (M)			ACG (T)	−0.039	7.81E-03	**AAG (K)**	**-0.467**	**1.36E-248**	AGG (R)	−0.179	1.90E-34
G	GUU (V)	0.346	4.56E-130	GCU (A)	0.132	1.55E-19	GAU (D)	0.338	3.48E-124	GGU (G)	0.052	3.99E-04
	**GUC (V)**	**-0.394**	**4.42E-171**	**GCC (A)**	**-0.441**	**9.31E-219**	**GAC (D)**	**-0.337**	**2.06E-123**	**GGC (G)**	**-0.249**	**2.88E-66**
	GUA (V)	0.309	1.48E-102	GCA (A)	0.311	1.99E-104	GAA (E)	0.416	6.46E-193	GGA (G)	0.150	1.44E-24
	GUG (V)	−0.095	1.17E-10	GCG (A)	0.045	2.14E-03	**GAG (E)**	**-0.416**	2.82E-193	GGG (G)	0.072	1.03E-06

a Bold identifies the synonymous codon in a codon family with the strongest significant negative correlation.

b Spearman’s correlation coefficient between frequency of a synonymous codon usage to the overall codon bias of the gene.

**Table 2:** Correlations between frequency of isoaccepting tRNA gene copy numbers and usage of synonymous codons and amino acids

	Synonymous Codon		Amino Acid	
Group	ρ*_Spearman_*	*P*-Value	ρ*_Spearman_*	*P*-Value
0	0.6520	6.913e-06	0.6924	0.00101
1	0.6719	2.824e-06	0.6310	0.00377
2	0.6122	3.448e-05	0.6994	0.00086
3	0.6874	1.338e-06	0.5985	0.00678
4	0.5785	1.145e-04	0.7100	0.00066
All	0.5934	6.856e-05	0.6836	0.00125

tRNA, transfer RNA.

### Identification of different codon usage groups

**Figure 2:**
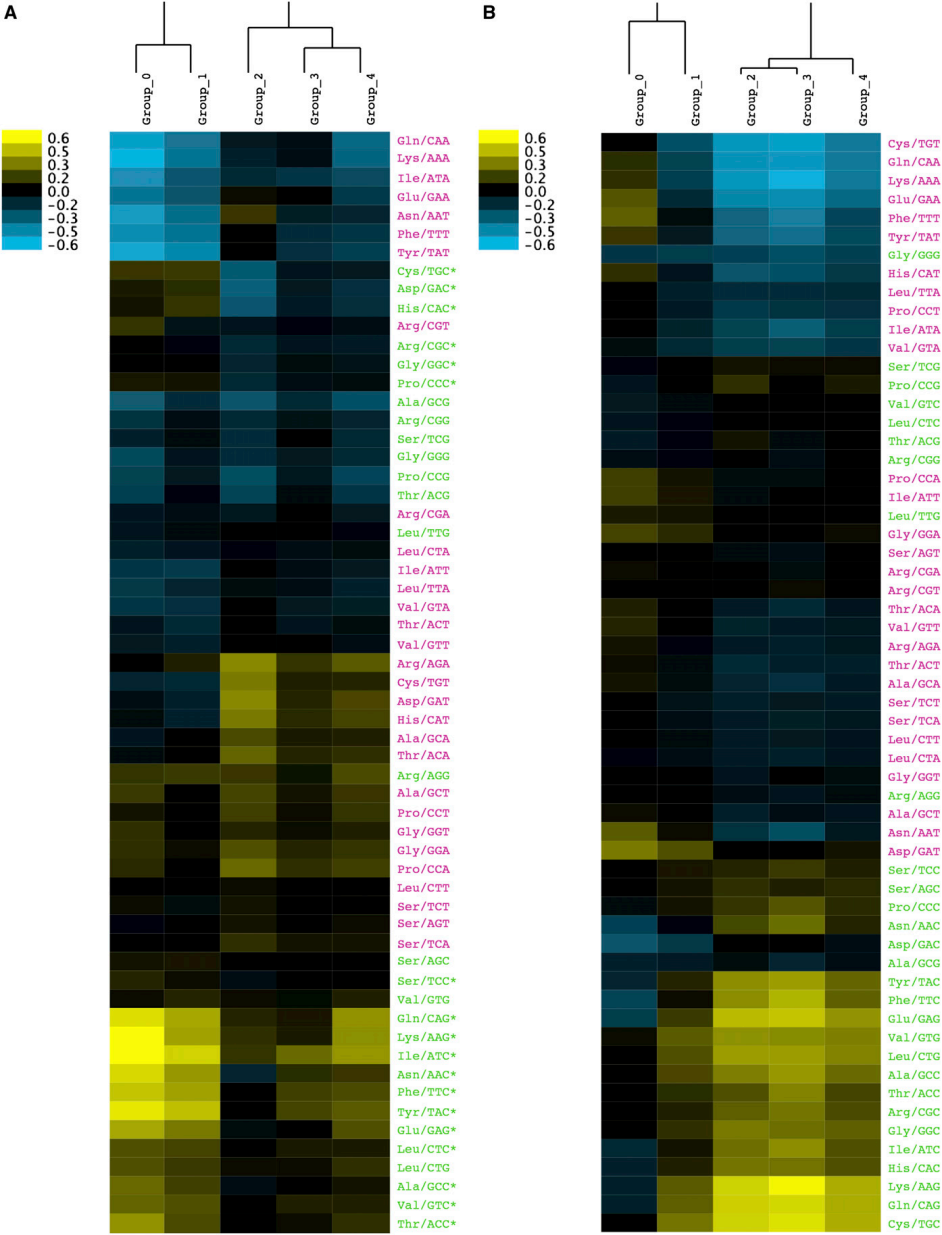
Heatmap of codon usage frequencies in the five codon usage groups (CUG) in (A) *Strongylocentrotus purpuratus* and (B) *Drosophila melanogaster*. Codon usage frequencies were centered on the equal usage expectation (*e.g.*, for fourfold degenerate codons, frequencies were centered on 1/4). Centered frequencies clustering was performed with hierarchical cluster analysis using pairwise complete-linkage by Euclidean distance for both columns (CUG) and rows (synonymous codons) using Cluster 3.0 (Eisen *et al.* 1998). Heatmap plots were generated with Java TreeView (Saldanha 2004). Synonymous codon labels are colored by the base composition of the third position (N3) with N3 of ‘A’ and ‘T’ nucleotides, colored pink, and ‘G’ and ‘C,’ colored green. The asterisk (*) denotes genome-wide preferred codons in *S. purpuratus* (see *Materials and Methods*).

The clustering method of Bailly-Bechet *et al.* (2006) identified five significantly distinct clusters of genes having different codon usage patterns (Table S3 and Figure 2A). The support for five groups was significantly better than six groups for both assignment probability [*B(S)* = 0.900 ± 0.002 *vs.* −0.880 ± 0.002, respectively] and maximum cluster stability [Δ*(S)* = 0.312 ± 0.003*vs*. 0.306 ± 0.002, respectively; see Figure S2A]. Table 3 shows that although codon bias was evident across all five groups (*i.e.*, GC3 was elevated above both GC*_i_* and GC*_f_*), it was most highly skewed in groups 0 and 1. Genes in groups 0 and 1 showed the highest levels of GC3 and the most extreme degrees of codon bias. Although groups 0 and 1 were similar in their levels of codon bias and patterns of synonymous codon usage (Table S3 and Figure 2A), group 0 genes exhibited dramatically greater levels of mRNA expression than the others (Table 3).

**Table 3:** Summary of codon bias, GC content, gene size, and mRNA expression levels in the five codon usage groups

Statistic	Group 0	Group 1	Group 2	Group 3	Group 4	All
No. of loci	396	861	1154	912	1300	4623
Mean Nc	49.72	54.54	53.57	57.63	54.65	54.46
Mean Nc′	42.50	47.21	54.74	53.93	50.87	51.02
Mean GC_cds_	0.521	0.517	0.450	0.481	0.484	0.484
Mean GC3	0.605	0.596	0.413	0.491	0.504	0.507
Mean GC_i_	0.353	0.356	0.345	0.356	0.350	0.351
Mean GC_f_	0.362	0.368	0.361	0.368	0.360	0.363
Mean number of codons	458.5	520.1	478.0	448.3	572.7	504.9
Mean number of exons	7.87	7.60	7.96	6.44	10.2	8.22
Mean transcript length, bp	9004	14,122	10,714	11,782	14,048	12,351
Mean mRNA expression (AU)	12,447.0	3051.0	3090.8	3142.2	3201.0	3926.0

mRNA, messenger RNA; AU, Arbitrary Units.

**Figure 3:**
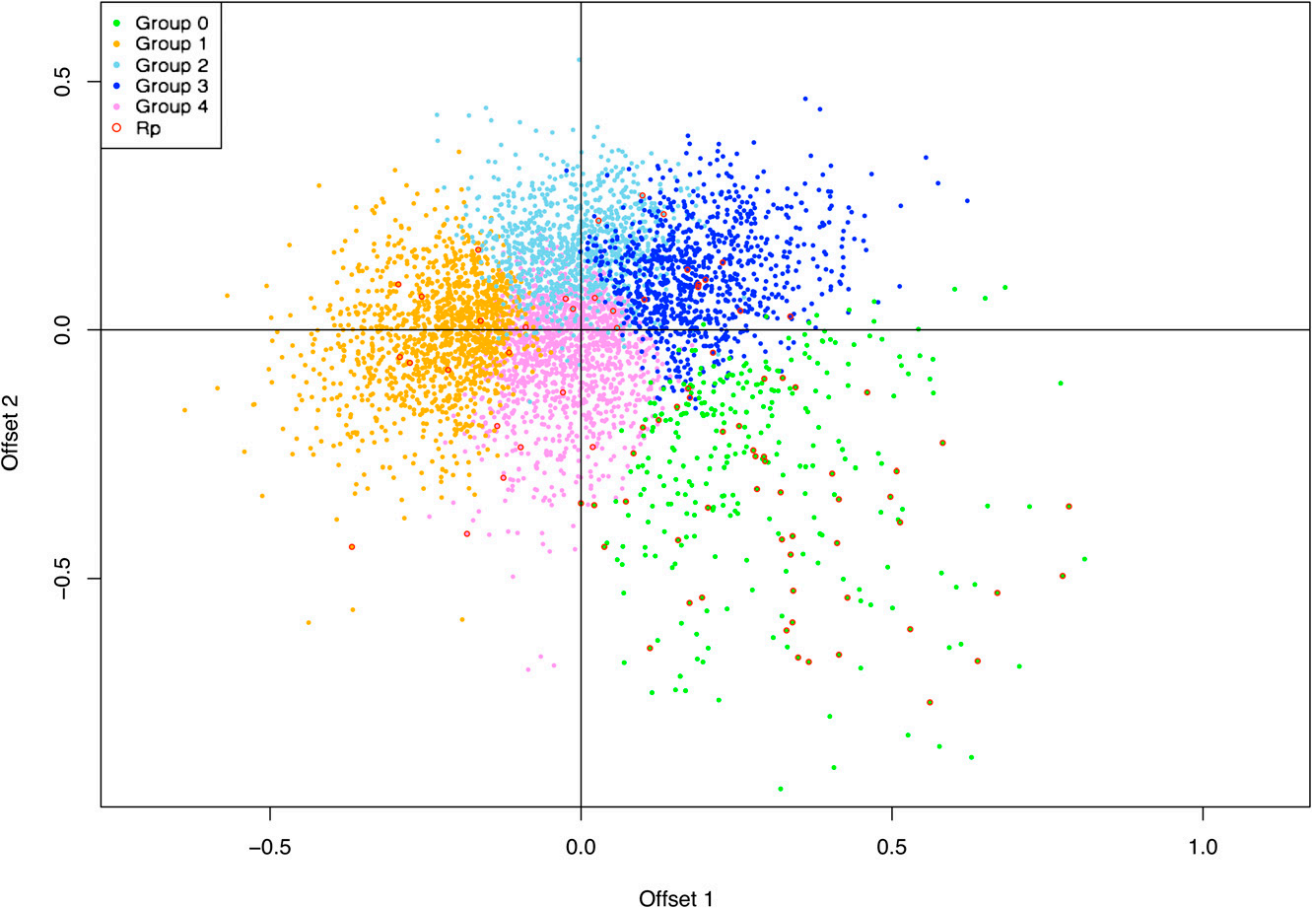
Plot of offsets β_1_ and β_2_ for a SCUMBLE model with four trends for *S. purpuratus* genes, colored by groups of genes clustered by codon usage distributions. Offset β_1_ is correlated most strongly to GC3 in all groups.

The SCUMBLE model of Kloster and Tang (2008) produced results that corroborated the existence of five distinct codon usage groups (Figure 3). Histograms of normalized variance (*NV*) suggested that at least four trends were required to explain the data (Figure S3A) and models with more than four trends failed to show improvement (Figure S3B). Offsets *β_1_* and *β_3_* correlated most strongly with GC3 and CT3 in all groups and in the pooled data, respectively (Table S4). Groups were differentiated, however, only by offset *β*_2_ that was most strongly associated with mRNA expression levels at 72 hr for group 0, GT3 in group 2, and CT3 in groups 1, 3, and 4 (Table S4).

### Patterns of codon usage bias

Codon bias in *S. purpuratus* was driven by synonymous codon usage in a subset of amino acids (Table 4). Four amino acids contributed significantly to codon bias in all five groups and in the pooled data [leucine (Leu), isoleucine (Ile), serine (Ser), and threonine (Thr)], whereas two amino acids did not contribute to codon bias in any group [histidine (His) and cysteine (Cys)]. Groups 0 and 1 exhibited the most strongly negative correlations (*i.e.*, *P* < 10^−10^) between sENC-X and Nc′ (9 each) and group 3 had the fewest (3). Although there was considerable variability in the contributions of individual amino acids across groups, the strongest correlations in the entire set of 4623 genes tended to show significant patterns in four or five of the groups.

**Table 4:** The contributions of individual amino acids to codon bias in *S. purpuratus*

	Spearman Correlation Coefficient Between sENC-X*^a^* and Codon Bias (Nc′)*^b^*					
Amino Acid	Group 0	Group 1	Group 2	Group 3	Group 4	All
Phe	−0.2817*	−0.1190*	−0.0349	−0.1389*	−0.0998*	−0.2320***
Leu	−0.4710***	−0.4230***	−0.2347***	−0.3133***	−0.3443***	−0.5338***
Ile	−0.3479***	−0.1645*	−0.1145*	−0.1926*	−0.2276***	−0.4752***
Val	−0.4540***	−0.2901***	−0.0887	−0.2167***	−0.2213***	−0.3698***
Ser	−0.2738*	−0.2942***	−0.2220***	−0.2015***	−0.1740*	−0.0881*
Pro	−0.1641	−0.1617*	−0.1618*	−0.0553	−0.0972*	0.0469
Thr	−0.4155***	−0.2503***	−0.2589***	−0.1756*	−0.2127***	−0.1869*
Ala	−0.3273***	−0.2561***	−0.1886***	−0.0902	−0.1108*	−0.0987***
Tyr	−0.3532***	−0.1159*	−0.0639	−0.1601*	−0.1461*	−0.2838***
His	−0.1075	−0.0884	0.0689	0.0327	0.0187	0.0528
Gln	−0.2788*	−0.1790*	−0.1207*	−0.0667	−0.1399*	−0.2867***
Asn	−0.3426***	−0.2335***	−0.0085	−0.1234*	−0.1298*	−0.2969***
Lys	−0.4319***	−0.2315***	−0.0601	0.0016	−0.1771***	−0.3864***
Asp	−0.0306	−0.1397*	0.0298	0.0013	0.0158	0.0567*
Glu	−0.2241*	−0.1997***	−0.0158	−0.0448	−0.1125*	−0.2452***
Cys	−0.0050	−0.0663	−0.0105	0.0565	−0.0315	0.0003
Arg	−0.3034***	−0.1641***	−0.3598***	−0.1693*	−0.2573***	−0.0451
Gly	−0.1303	−0.1055	−0.0958	−0.0003	−0.1383*	−0.0213

sENC-X, scaled ENC-X; Phe, phenylalanine; Leu, leucine; Ile, ; isoleucine; Val, valine; Ser, serine; Pro, proline; Thr; threonine; Ala, alanine; Tyr, tyrosine; His, histidine; Gln, glutamine; Asn, asparagine; Lys, lysine; Asp, aspartic acid; Glu, glutamine; Cys, cysteine, Arg, arginine; Gly, glycine. *Significance at *P* < 0.001. ***Significance at *P* < 10^−10^

a Moriyama and Powell 1997.

b Novembre 2002.

**Figure 4:**
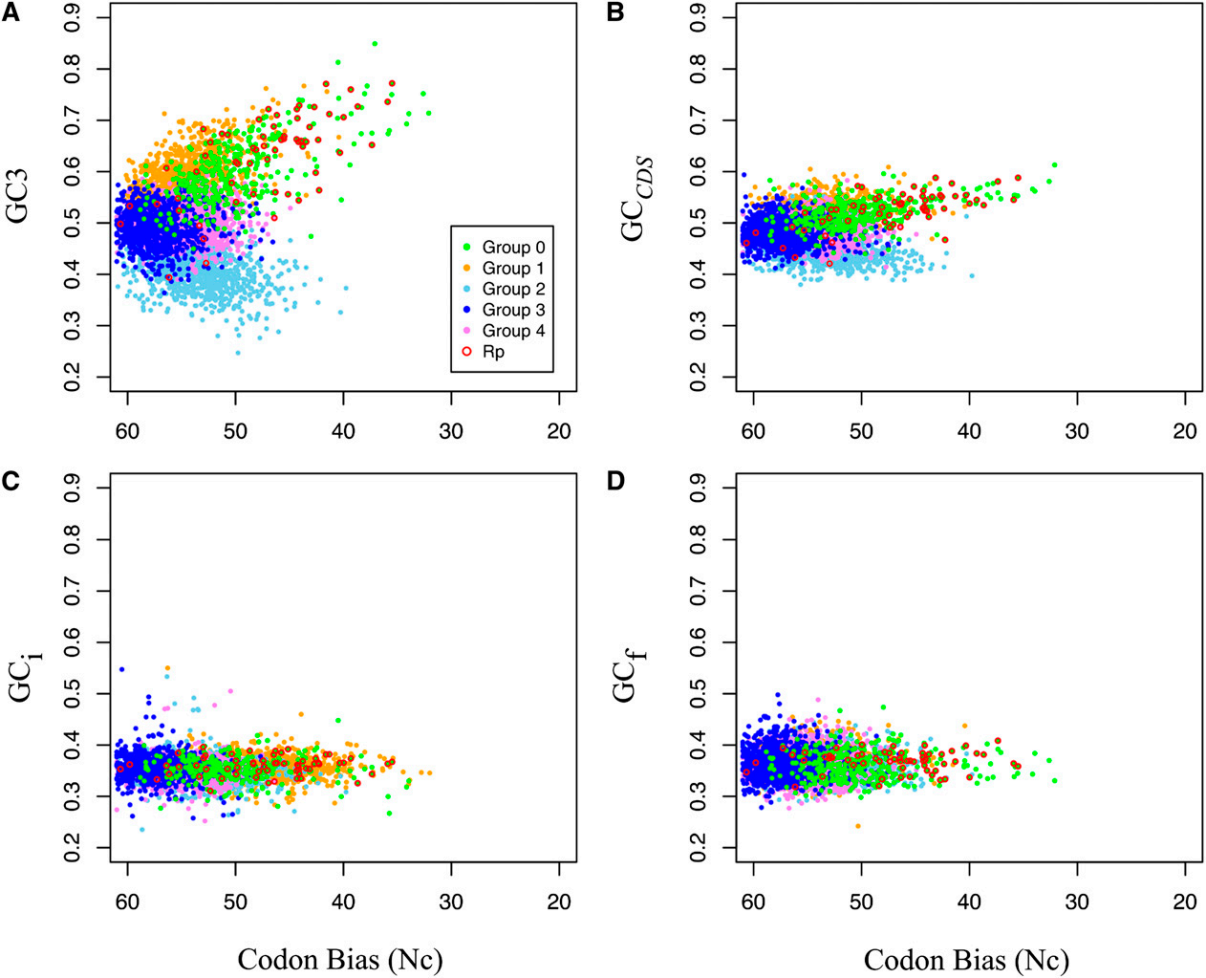
Codon bias and GC composition in protein coding gene regions for different codon usage groups. Codon bias is represented as Wright’s Nc (Wright 1990). Regional GC composition is calculated from (A) GC3 content of exons, (B) GC content of exons, (C) GC content of introns, and (D) GC content of flanking regions. “Rp” denotes annotated ribosomal proteins. The genome-wide mean GC content is 36.9%.

The GC content of third positions (GC3) and coding regions (GC_cds_) exhibited significant correlations with the degree of codon bias (Figure 4, A and B and Table 5). By contrast, there was a limited association between the GC content of introns and flanking regions and the degree of codon bias (Figure 4, C and D and Table 5). Our results show that GC content of introns or flanking regions for all genes displayed weak associations with Nc (Table S5) and no significant correlations with Nc′ (Table 4). GC3 was strongly correlated with GC_cds_ in all groups (Table 5). Relationships between the GC content of introns and flanking regions with both GC3 and GC_cds_ were much weaker and inconsistent among groups (Table 5).

**Table 5:** Correlations between codon bias (Nc′), GC composition, and rates of protein evolution in *S. purpuratus*

		Spearman’s Correlation Coefficient for Each Group*^a^*					
Variable 1	Variable 2	Group 0	Group 1	Group 2	Group 3	Group 4	All
Codon bias							
Nc′	GC3	−0.7049***	−0.6316***	−0.2946***	−0.432***	−0.4489***	−0.7325***
Nc′	GC_cds_	−0.4557***	−0.2381***	−0.0668***	−0.187***	−0.1392***	−0.5584***
Nc′	GC_i_*^b^*	0.0279	0.0919	0.0933	0.2226***	0.1315*	−0.0195
N′	GC_f_	0.103	0.0648	0.1394*	0.0547	0.0917*	0.0469
Regional GC composition							
GC3	GC_cds_	0.4811***	0.3884***	0.3299***	0.3872***	0.2443***	0.7409***
GC3	GC_i_	0.0837	0.1491*	0.1259*	0.054	0.1021*	0.2218***
GC3	GC_f_	0.0548	0.0774	0.0424	0.0889	0.0605	0.0872*
GC_cds_	GC_i_	0.1576	0.1009	0.0761	0.0619	0.1076*	0.2073***
GC_cds_	GC_f_	0.0913	0.0377	0.0063	0.0951	0.024	0.0737*
GC_i_	GC_f_	0.1221	0.084	0.1113*	0.1109	0.1383*	0.1333***
Rate comparisons*^c^*							
Nc′	*dS*	−0.124	−0.1538*	−0.0552	−0.0243	−0.0389	−0.1541***
Nc′	*dN*	0.1682	0.18249*	0.0577	0.126	0.1475*	0.1571***
Nc′	*dN/dS*	0.2231	0.2292*	0.0667	0.133	0.1515*	0.1993***

* Significance at *P* < 0.001. *** Significance at *P* < 10^−10^

a Number of genes in each group: all (4623), cluster 0 (396), cluster 1 (861), cluster 2 (1154), cluster 3 (912), cluster 4 (1300).

b Number of genes with introns for each group: all (4389), cluster 0 (368), cluster 1 (814), cluster 2 (1113), cluster 3 (826), Cluster 4 (1268).

c Number of genes with comparative data for each group: all (2954), cluster 0 (225), cluster 1 (593), cluster 2 (744), cluster 3 (563), cluster 4 (829).

**Figure 5:**
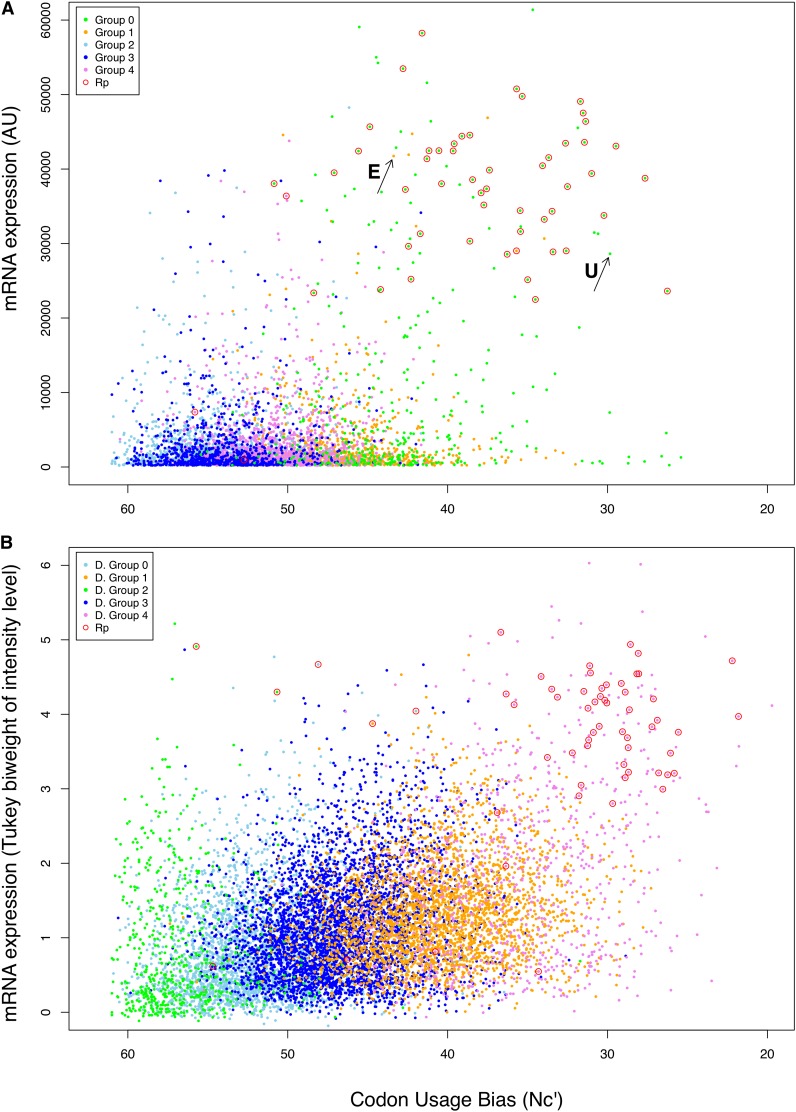
Synonymous codon usage bias and mRNA expression levels for genes in different codon usage groups in (A) *S. purpuratus* and (B) *Drosophila melanogaster*. Codon usage bias is Novembre’s Nc′. Red circles surround annotated ribosomal proteins (‘Rp’). The black arrow labeled “U” points to “ubiquitin-like/S30 ribosomal fusion protein” (SPU_005280), an example of a highly expressed gene in *S. purpuratus*. The black arrow labeled “E” points to Sp-Ets1/2 (SPU_002874), an example of a highly expressed, highly biased gene that does not belong to codon usage group 0.

The magnitude of codon bias was significantly associated with gene expression levels, but only in group 0 (Nc’ ρ = −0.2578, *P* < 0.0001; Nc ρ = −0.2695, *P* < 0.0001). Highly biased, highly transcribed genes were primarily found in group 0 (Figure 5A). For example, 51 of the 54 annotated ribosomal proteins were assigned to group 0 (of the remaining three, one was in group 1 and two in group 4). However, as with *Notch* in *Drosophila*, we observed highly expressed, highly biased genes that did not share a synonymous codon usage pattern with ribosomal proteins and the other members of group 0. For example, Sp-Ets1/2 (SPU_002874) is a member of the ETS family of transcription factors. It acts as a transcriptional activator or repressor and is involved in cell senescence and death, stem cell development and tumorigenesis (RefSeq Release 49, July 2011). Sp-Ets1/2 is highly expressed (41,923 AU) and highly biased (Nc′ = 42.443) but is a member of group 1 rather than group 0.

Gene length was also negatively correlated with mRNA expression and Nc′ (Table 6, Figure S4). We did not observe relationships between codon bias and position along the protein using intragenic spatial codon usage estimates (Qin *et al.* 2004). The magnitude of codon bias did not differ between the middle sections of genes relative to amino- or carboxy-terminal regions, did not change over the length of the gene for different size classes and was not correlated with intron number or length (not shown). Thus, we were unable to find support for interference selection acting on synonymous codon usage in *S. purpuratus* (Comeron and Kreitman 2002).

**Table 6:** Correlations between gene size and mRNA expression, codon bias (Nc′), and GC content in *S. purpuratus*

		Spearman’s Correlation Coefficient for Each Group*^a^*					
Variable 1	Variable 2	Group 0	Group 1	Group 2	Group 3	Group 4	All
Coding and transcript lengths							
N_codons_	mRNA level*^b^*	−0.2305*	−0.1052	−0.1366*	−0.0823	−0.0605	−0.1164***
N_codons_	Nc′	0.1991*	0.2118*	0.0559	0.2119*	0.1226*	0.0649*
N_codons_	GC_cds_	−0.1058	−0.1738*	0.1404*	0.0106	−0.0558	0.0345
N_exons_	mRNA level	−0.1316	−0.0437	−0.0346	−0.0578	−0.0315	−0.0507*
T_length_	mRNA level	−0.1379	−0.0521	−0.0128	−0.0354	−0.0233	−0.0574*
T_length_	Nc′	0.1055	−0.0697	−0.0915	−0.112*	−0.0412	−0.049*

mRNA, messenger RNA. *Significance at *P* < 0.001. ***Significance at *P* < 10^−10^.

a Number of genes in each group: all (4623), cluster 0 (396), cluster 1 (861), cluster 2 (1154), cluster 3 (912), cluster 4 (1300).

b log_10_(maximum observed expression in AU).

### Conservation of preferred codons

We tested whether preferred codons were more evolutionarily constrained by applying Akashi’s (1994) method. The conservation of codons was assessed using alignments containing *S. purpuratus*, *A. fragilis*, and *S. franciscanus*. We observed that preferred codons were more likely to be conserved than unpreferred codons in all five groups and for all genes combined (Table 7). This is remarkable considering that each group had a different set of preferred codons defined for the test. The Woolf test on homogeneity of odds ratios (Woolf 1955) showed significant three-way associations for all groups. To further test this result, we randomly generated sets of preferred codons and found that our previously identified list exhibited a significantly stronger association between codon preference and conservation across each (Table 7). In all five codon usage groups, stronger correlations were detected between our identified preferred codons and the evolutionarily conserved codons than that for the all genes combined.

**Table 7:** Akashi’s test for the conservation of preferred codons between species

Group	No. of Genes	M-H Χ^2^	OR*^a^*	*P*-value	P(Better Codon Set)*^b^*
0	225	31.46	1.287	1.02e-08	< 0.001
1	592	38.46	1.163	2.70e-10	0.005
2	744	36.25	1.098	8.69e-10	0.007
3	562	24.08	1.137	4.61e-10	< 0.001
4	828	123.84	1.213	4.59e-29	0.024
All	2349	247.77	1.193	3.98e-56	0.036

OR, odds ratio.

a Woolf test on homogeneity of ORs (Woolf 1955) shows significant three-way association for all groups.

b P(Better Codon Set) is the fraction of 1000 randomly generated alternate preferred synonymous codon sets having a stronger association with conserved codons than the observed preferred set (see *Materials and Methods*).

### Role of preferred codons in stabilizing RNA structure

We found that the third positions of preferred codons were significantly more likely to be paired in stems within folded RNA secondary structures in groups 2 and 3 (Table 8). Although all five groups had different distributions of preferred third positions (Table S2), each had two N3 preferred codons that were significantly overrepresented in stem pairs. In all five groups, G3 was significantly associated to be paired in stems in preferred codons. C3 of preferred codons were more likely to be stem-paired in all groups except group 2, where it was replaced by U3. The identity of the majority codon N3 base did not match the preferred stems for any group. The Woolf test on homogeneity of odds ratios (Woolf 1955) produced significant 3-way associations for all groups.

**Table 8:** Tests for stem-pairing of preferred codon N3 in mRNA secondary structure predictions

	M-H Χ^2^*^a^*				
Group	A3	C3	G3	U3	N3
0	18.83	261.80***	19.00*	295.36	112.31
1	987.55	612.26***	164.31***	n/a	670.40
2	1810.24	2.02	254.02***	944.23***	4224.29***
3	806.18	242.88***	358.13***	1.69	3011.43***
4	2355.78	311.50***	536.99***	6.14	1.16
All	n/a	1945.18***	1594.94***	n/a	3675.23

mRNA, messenger RNA. *P < 0.01. ***P < 1e-10.

a Mantel-Haenszel Χ^2^ test with continuity correction and an alternate hypothesis that preferred codons are more likely to be a stem than loop in mRNA secondary structure.

### Comparison to codon usage patterns in *Drosophila*

To determine whether similar clusters of codon usage patterns occur in *Drosophila melanogaster*, we applied the Bailly-Bechet *et al.* (2006) clustering method to 11,223 gene models obtained from flybase.org. Similar to our results for *S. purpuratus*, we detected five significantly distinct groups (Figure 2B, Figure S2B). Although similar in overall number, the patterns of codon usage differed between the species. Overall, 2675 (23.8%) genes were assigned to group 0, 2919 (26.0%) to group 1, 607 (5.4%) to group 2, 4156 (37.0%) to group 3, and 867 (7.7%) to group 4. Highly expressed, highly biased genes were almost entirely represented by *Drosophila* group 4 (Figure 5B). As found in *S. purpuratus*, a majority of annotated small and large subunit ribosomal proteins were observed in this highly expressed group (54 of 60). Third positions in *Drosophila* groups 1, 2, 3, and 4 were more likely to be G or C (Figure 2B). Conversely, N3 for genes in *Drosophila* Group 2 were more likely to be A or T. Despite being highly biased (*Nc*′ = 43.31) and typically highly expressed, the synonymous codon usage in *Notch* was more similar to other members of *Drosophila* Group 3. Genome-wide, *S. purpuratus* genes were not as biased as *D. melanogaster* (Nc′ = 46.04 ± 6.83 and 51.08 ± 5.08, respectively). *S. purpuratus* group 0 genes were more biased than *D. melanogaster* genome-wide average (Nc′ = 42.50 ± 15.5) but less biased than *D. melanogaster* group 4 (Nc′ = 39.08 ± 7.51).

## Discussion

Synonymous codon usage is influenced by the competing actions of random genetic drift, natural selection, and mutation (Sharp *et al.* 1995; Duret 2002; Hershberg and Petrov 2008). Despite decades of study on codon bias the relative contributions of mutational bias *vs.* selection favoring the speed and accuracy of protein translation, translational robustness, or mRNA stability remains unclear (Duret 2002; Hershberg and Petrov 2008; Plotkin and Kudla 2011). Selection for translational efficiency has long been recognized as an important factor affecting synonymous codon usage bias in unicellular and multicellular organisms (*e.g.*, Powell and Moriyama 1997; Akashi 1994). However, other forms of selection can favor translationally suboptimal codons (Hershberg and Petrov 2008) that in competition with translational selection can result in heterogeneous patterns of codon usage across the genome (Akashi and Eyre-Walker 1998). The present study has confirmed that multiple distinct patterns of codon usage bias exist in *S. purpuratus* that appear to result from different modes of selection (*i.e.*, translational efficiency and mRNA stability). Our results also suggest that mutational bias has played a negligible role in generating codon bias in *S. purpuratus*.

### Distinct codon usage groups occur in *S. purpuratus*

Synonymous codon usage varies between genes varies in both composition and magnitude (Sharp *et al.* 1986; Shields and Sharp 1987). Genome-wide patterns of codon bias are driven primarily by the most highly biased genes, which, if they share preferred codons, will dominate the synonymous codon usage patterns. It is thus easy to overlook more subtle patterns of codon bias. This is evident in yeast where codon usage differs between highly and lowly expressed genes in both the level and nature of the bias (Sharp *et al.* 1986). A similar situation is seen in *Bacillus subtilis* where correspondence analysis identified an axis with ribosomal proteins at one end and genes with different codon usage patterns at the other end (Shields and Sharp 1987). *Notch* is a highly expressed and highly biased gene in *Drosophila* but exhibits a codon usage distribution different from other similar loci (Dumont *et al.* 2004; Singh *et al.* 2007). These subtle patterns of codon usage appear to be biologically significant and need to be considered in studies examining codon bias.

We identified five distinct codon usage groups in *S. purpuratus* using two independent methods (Figures 2A and Figure 3). The magnitude of codon bias and the usage of genome-wide preferred codons varied dramatically between groups (Table 1, Table 3 and Table S3), as did the mean levels of maximum mRNA expression during early development (Figure 5A). Previously, the detection of distinct codon usage clusters has been restricted to bacteria and yeast (*e.g.*, Bailly-Bechet *et al.* 2006; Kloster and Tang 2008). In *E. coli* K12 and *B. subtilis*, Bailly-Bechet *et al.* (2006) identified four and five distinct codon usage clusters, respectively. In both species, genes belonging to different clusters were associated together in different chromosomal regions implicating some role for translational selection. The present study represents the first documentation of similar codon usage clusters existing in a metazoan. To assess the generality of our results in *S. purpuratus* we also identified five distinct codon usage groups in *D. melanogaster* (Figure 2B), a well studied species where translational selection on rate and accuracy has been previously documented (Powell and Moriyama 1997). *Notch*, being an interesting anomaly for synonymous codon usage in *Drosophila*, was expected to have a different codon usage pattern from other highly expressed and highly biased genes. As expected, *Notch* had a different codon usage distribution from the majority of ribosomal proteins in *Drosophila*. Our identification of distinct codon usage groups in *D. melanogaster* supports the reliability of the patterns we observed in *S. purpuratus* and suggests that similar clusters may be present in other species.

### Mutational processes cannot explain codon bias in *S. purpuratus*

If mutational bias is responsible for generating codon usage bias, synonymous sites are expected to have nucleotide compositions similar to introns and flanking regions. *S. purpuratus* is an excellent species to test for mutational bias because it possesses an AT-biased genome lacking isochores and the majority of genes reside in regions of GC content between 35 and 39% (Sodergren *et al.* 2006). Despite this overall AT bias, all 18 preferred codons used a C or a G at their third positions. In our data, the mean GC content of introns and flanking regions were similar to the genome-wide average and did not vary among the different codon usage clusters (Table 3). The magnitude of codon bias exhibited strong correlations with mean GC3 and GC*_cds_* in the most highly biased groups (0 and 1). However, the degree of codon bias in groups 0 and 1 showed no associations with either GC_i_ or GC_f_ (Table 5, Table S5, and Figure 4). These observations suggest that mutational bias has played a negligible role in influencing patterns of codon bias in *S. purpuratus*.

### Selection on translation drives major codon usage patterns

Codon usage and tRNA abundances are thought to have coevolved to maximize the speed (Bulmer 1987) or the accuracy of protein translation (Akashi 1994). Speed of translation is an adaptation for translation rate whereas translational accuracy acts to minimize the incorporation of incorrect amino acids into proteins. These two interrelated drivers of selection on translational efficiency (speed and accuracy) are difficult to disentangle and share similar predictions. For example, both hypotheses predict that the abundances of tRNA species should correlate with preferred codons in highly expressed genes (Ikemura 1985) and that high levels of gene expression should correlate with high levels of codon bias (Duret and Mouchiroud 1999). In *S. purpuratus* we obtained evidence for both of these predictions. We found that tRNA gene frequencies were significantly correlated with genome-wide preferred codons and tRNA gene copy number exhibited a significant positive relationship with amino acid counts. Furthermore, the magnitude of codon bias was strongly related to mRNA expression levels during early development (Table 3 and Figure 5A).

Although we detected genome-wide evidence for translational selection, it was driven exclusively by genes in group 0 that represented only 8.6% of all loci. The ability of such a small number of genes to drive this overall pattern is attributable to their dramatically greater levels of mRNA expression (roughly four times the other groups; see Table 3). Strikingly similar results were obtained for *D. melanogaster*, in which highly expressed and highly biased genes were represented in only one of the five clusters identified (group 4; Figure 5B). In both species, the vast majority of ribosomal protein genes were present in the highly expressed cluster and *S. purpuratus* group 0 was overrepresented with GO terms related to transcription and cell growth (Table S6). Translational selection thus apparently acts on only one of the five codon usage groups identified in both species and only a small proportion of genes (<10%) are driving the genome-wide correlations between codon bias and mRNA expression.

In *S. purpuratus*, codon usage group 1 shares similar patterns of codon preference to group 0 but fails to exhibit a correlation with gene expression levels. There are three amino acids [serine (Ser), proline [Pro], and glycine [Gly]) with different preferred codons between groups 0 and 1 (Table 4). Only Ser contributes to codon bias in both groups (Table S3), whereas Pro contributes only to group 0. If group 1 genes have also been selected for translational efficiency then one explanation for this anomalous result is they are not expressed during early development but are highly expressed at later developmental stages. Recent studies in *Drosophila* have found that larval stages exhibit greater overall codon bias than adult stages, supporting the hypothesis that codon usage bias is influenced by selection for efficient protein production (Vicario *et al.* 2008). In contrast, genes expressed after embryogenesis in *C. elegans* have greater synonymous substitution rates than those expressed during embryogenesis (Castillo-Davis and Hartl 2002). The possibility of translational selection acting on group 1 genes in *S. purpuratus* at later developmental stages needs to be investigated further.

### Preferred codons are evolutionarily conserved

Selection for the accuracy of translation is an additional explanation for the relationship between codon bias and rates of translation in *Drosophila* (Akashi 1994). The model assumes that the misincorporation of amino acids due to errors in translation adversely affects the functionality of proteins and reduces cell fitness. There are two unique predictions of the translational accuracy model: (1) synonymous codon usage at evolutionary conserved sites will be biased toward preferred codons, and (2) the degree of conservation will be stronger in highly expressed genes. As found for *Drosophila*, we observed that preferred codons were evolutionarily conserved across all genes in *S. purpuratus* (Table 7). In addition, we observed that preferred codons were conserved to a similar degree in all five codon usage groups with their respective sets of preferred codons despite differences in their synonymous codon usage patterns, levels of mRNA expression, and their magnitude of codon bias (Table 7). This is particularly noteworthy for group 2, whose preferred codons end primarily in A and T. Selection for translational accuracy should also be strongest in the most highly expressed group 0 but instead appeared to be independent of mRNA expression. Therefore, selection for translational accuracy cannot account for the different patterns of codon usage observed between the clusters.

### Preferred third codon positions are more likely to pair in mRNA stems

Selection to optimize heteroduplex base paring and maximize the stability of mRNA secondary structure has also been considered as a potential driver of codon usage bias for many years (Fitch 1974; Klämbt 1975; Grantham *et al.* 1980). A relationship between folding stability and codon preference has been established through the examination of mRNA secondary structure in mammals and other groups (Fitch 1974; Hasegawa *et al.* 1979; Chamary and Hurst 2005; Meyer and Miklós 2005; Chamary *et al.* 2006; Shabalina *et al.* 2006). mRNA stability is expected to be influenced by base composition at third positions (N3) (Carlini *et al.* 2001). A preference for G or C in N3 is seen in the most highly biased genes of *Drosophila*, which is almost entirely explained by an increased use of C over G (Shields *et al.* 1988). This preference for C in N3 of highly biased loci is not easily explained by local mutation effects but may result from its effect on mRNA stability (Gillespie 1991).The stability of mRNA secondary structure can be increased through GC heteroduplex pairing in N3 or by an increase in the number of heteroduplex pairs. Recognizing there are other competing processes that determine preferred N3 composition (Chamary and Hurst 2005), and different definitions of preference not limited to GC (this study), we restricted our analysis to stems and loops and tested if the third positions of preferred codons were more likely to reside as paired members of stems than unpaired members of loops. More specifically, if natural selection acts on synonymous codons to increase mRNA stability then we expect N3 of preferred codons to more likely to reside as paired members of stems than unpaired members of loops.

In *S. purpuratus*, we observed that any nucleotide in the third position of preferred codons (N3) were more likely to be in paired in stems than unpaired in loops only for genes in codon usage groups 2 and 3 (Table 8). Ignoring codon usage groups, the Mantel-Haenszel tests were significant for C3 and G3, which is consistent with the observation that all genome-wide preferred codons had a G or C in their 3^rd^ positions. Considering the groups separately, group 2 differed sharply from the others in having G3 or U3 of preferred codons pairing in stems over loops. Group 0 exhibited the weakest relationships, which is not surprising because selection for mRNA stability may conflict with translational selection and other processes. No significant relationships were observed between mRNA stability and expression levels. Here again, mRNA stability can impact rates of translation and highly stable mRNA structures may be specifically avoided in rapidly transcribed regions of genes (Mita *et al.* 1988; Zama 1990). Overall these results implicate a role for mRNA folding, perhaps acting in a compensatory fashion, in determining the patterns of synonymous codon usage in *S. purpuratus*.

### Codon bias decreases with gene length

Codon bias decreases with increasing gene length in *S. purpuratus* similar to that observed in other species (*e.g.*, Comeron *et al.* 1999; Duret and Mouchiroud 1999; Ingvarsson 2007; Qiu *et al.* 2011). This decrease in codon bias with increasing gene length may be a side-effect of the economy of translation where highly expressed, highly biased genes tend to be short (Akashi 2003). Indeed, in group 0 gene length was correlated with both codon bias and mRNA expression levels, further supporting the action of translational selection on this group. However, in the other codon usage groups gene length was correlated with either codon bias (groups 1, 3, and 4) or expression (group 2), but not both. The negative correlation between gene length and codon usage may also be due to GC-biased gene conversion (Stoletzki 2011). Our results do not support an alternative prediction for translational accuracy where the cost of incorporation of erroneous amino acids during elongation will drive large genes to have higher codon bias than small genes (Eyre-Walker 1996) as observed in yeast (Eyre-Walker 1996; Moriyama and Powell 1998; Stoletzki and Eyre-Walker 2007).

In summary, we detected significant nonrandom patterns of synonymous codon usage in the purple sea urchin that were incompatible with the predicted effects of mutational bias. Five distinct clusters of genes were identified that used different sets of preferred codons. However, only one cluster exhibited a correlation between codon bias and mRNA expression consistent with translational selection. The third positions of preferred codons were more likely to be paired in stems in folded mRNA secondary structures in two additional codon usage groups, implicating selection for mRNA stability on synonymous codon usage. The discovery of five distinct codon usage groups in *D. melanogaster* was similar to that found for *S. purpuratus*, but the patterns of codon usage differed between species. However in both species only one cluster representing <10% of all genes was associated with translational selection. Our results suggest that other forms of selection might be more important in determining the overall patterns of synonymous codon usage and that similar codon usage clusters might be present in other groups.

## Supplementary Material

Supporting Information
