# Supplementary material for: Genome-Wide Patterns of Codon Bias Are Shaped by Natural Selection in the Purple Sea Urchin, Strongylocentrotus purpuratus
Source: G3 (Bethesda). 2013 Jul 1;3(7):1069–83. doi: 10.1534/g3.113.005769 (PMC3704236; doi:10.1534/g3.113.005769)
Supplement: Supporting Information [file supp_3_7_1069__index.html]

Genome-Wide Patterns of Codon Bias Are Shaped by Natural Selection in the Purple Sea Urchin, Strongylocentrotus purpuratus — Supporting Information 

# Genome-Wide Patterns of Codon Bias Are Shaped by Natural Selection in the Purple Sea Urchin, *Strongylocentrotus purpuratus*

## Supporting Information for Kober and Pogson, 2013

**Files in this Data Supplement:**

- Supporting Information - Figures S1-S4, Files S1-S2, and Tables S1-S6 (PDF, 1 MB)
- Figure S1 - Genome-wide preferred codon usage for Ile (PDF, 928 KB)
- Figure S2 - The cluster stability curves for (A) *Strongylocentrotus purpuratus* and (B) *Drosophila melanogaster* (PDF, 99 KB)
- Figure S3 - Normalized variance for SCUMBLE models with up to 10 trends in *S. purpuratus* (PDF, 105 KB)
- Figure S4 - Coding sequence (CDS) length scatterplots (PDF, 860 KB)
- Table S1 - 2x2 contingency table for a synonymous codon preference and mRNA secondary structure for a given gene (PDF, 45 KB)
- Table S2 - Counts of preferred N3 for each group by preference method (PDF, 65 KB)
- Table S3 - Synonymous codon usage in *S. purpuratus* (PDF, 99 KB)
- Table S4 - The strongest and most significant Spearman correlation coefficients for each of the first three SCUMBLE offsets for a SCUMBLE model with 4 trends (PDF, 63 KB)
- Table S5 - Correlations between codon bias (Nc), regional GC content and rates of protein evolution in *Strongylocentrotus purpuratus* (PDF, 77 KB)
- Table S6 - Significantly Over-Enriched Gene Ontology (GO) codes for *S. purpuratus* gene groups (PDF, 86 KB)
- File S1 - *D. melanogaster dataset* (.txt, 15 MB)
- File S2 - *S. purpuratus* dataset (.txt, 6 MB)
