# Supplementary material for: Genome-Wide Patterns of Codon Bias Are Shaped by Natural Selection in the Purple Sea Urchin, Strongylocentrotus purpuratus
Source: G3 (Bethesda). 2013 Jul 1;3(7):1069–83. doi: 10.1534/g3.113.005769 (PMC3704236; doi:10.1534/g3.113.005769)
Supplement: Supporting Information [file supp_g3.113.005769_005769SI.pdf]

G3·Genes | Genomes | Genetics

Genome-wide patterns of codon bias are shaped by natural selection in the purple sea urchin, *Strongylocentrotus purpuratus*

Kord M. Kober [*,§,1] and Grant H. Pogson [*]

[*] Department of Ecology & Evolutionary Biology, University of California, Santa Cruz, CA 95064, USA, [§] Department of Physiological Nursing, University of California San Francisco, San Francisco, CA 94143, USA

[1] Corresponding author: Dept. of Physiological Nursing, 2 Koret Way, #N-631, San Francisco, CA 94143-0610, Email: kord.kober@nursing.ucsf.edu

DOI: 10.1534/g3.113.005769

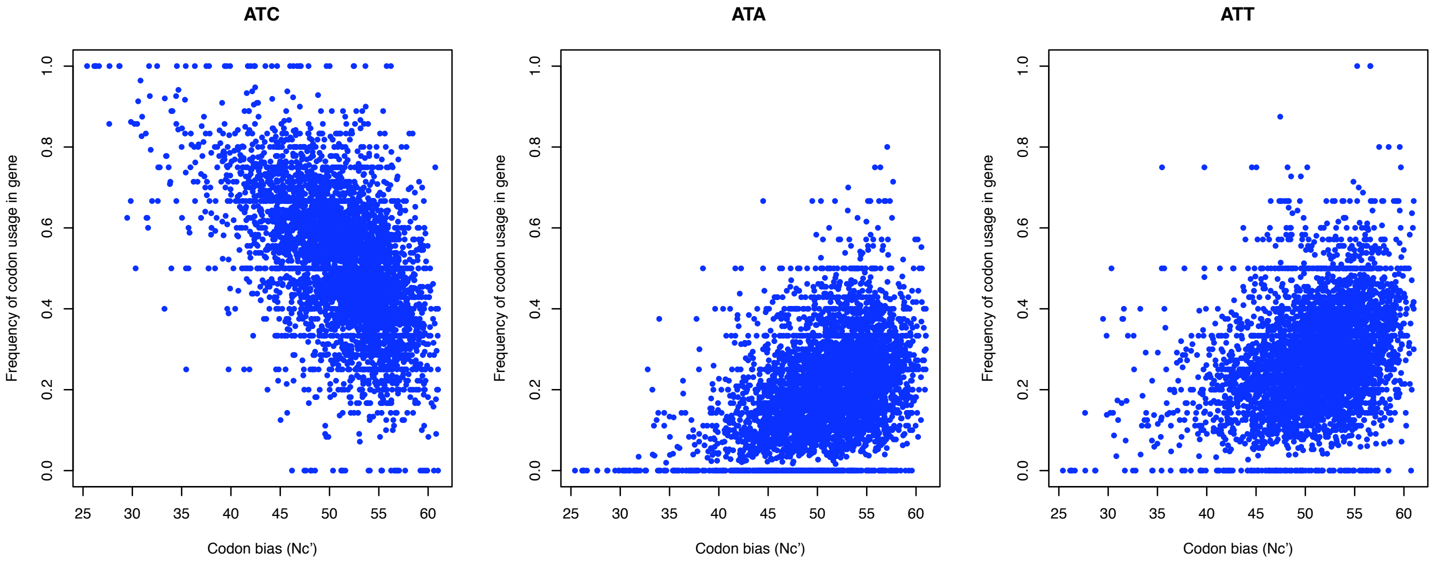

Figure S1 Genome-wide preferred codon usage for Ile.

A

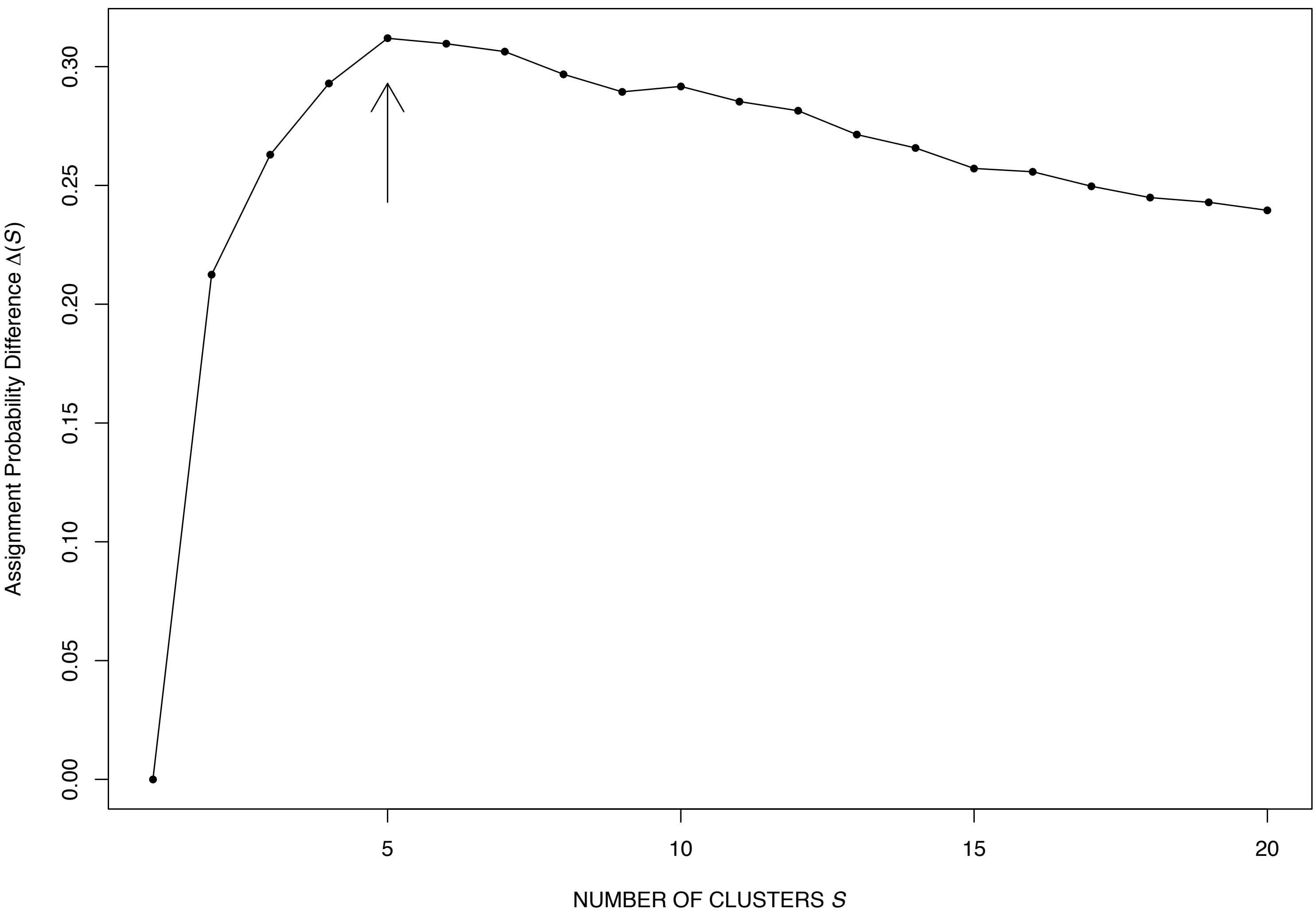

Assignment Probability Difference Δ(*S*)
0.00
0.05
0.10
0.15
0.20
0.25
0.30
5
10
15
20
NUMBER OF CLUSTERS *S*

B

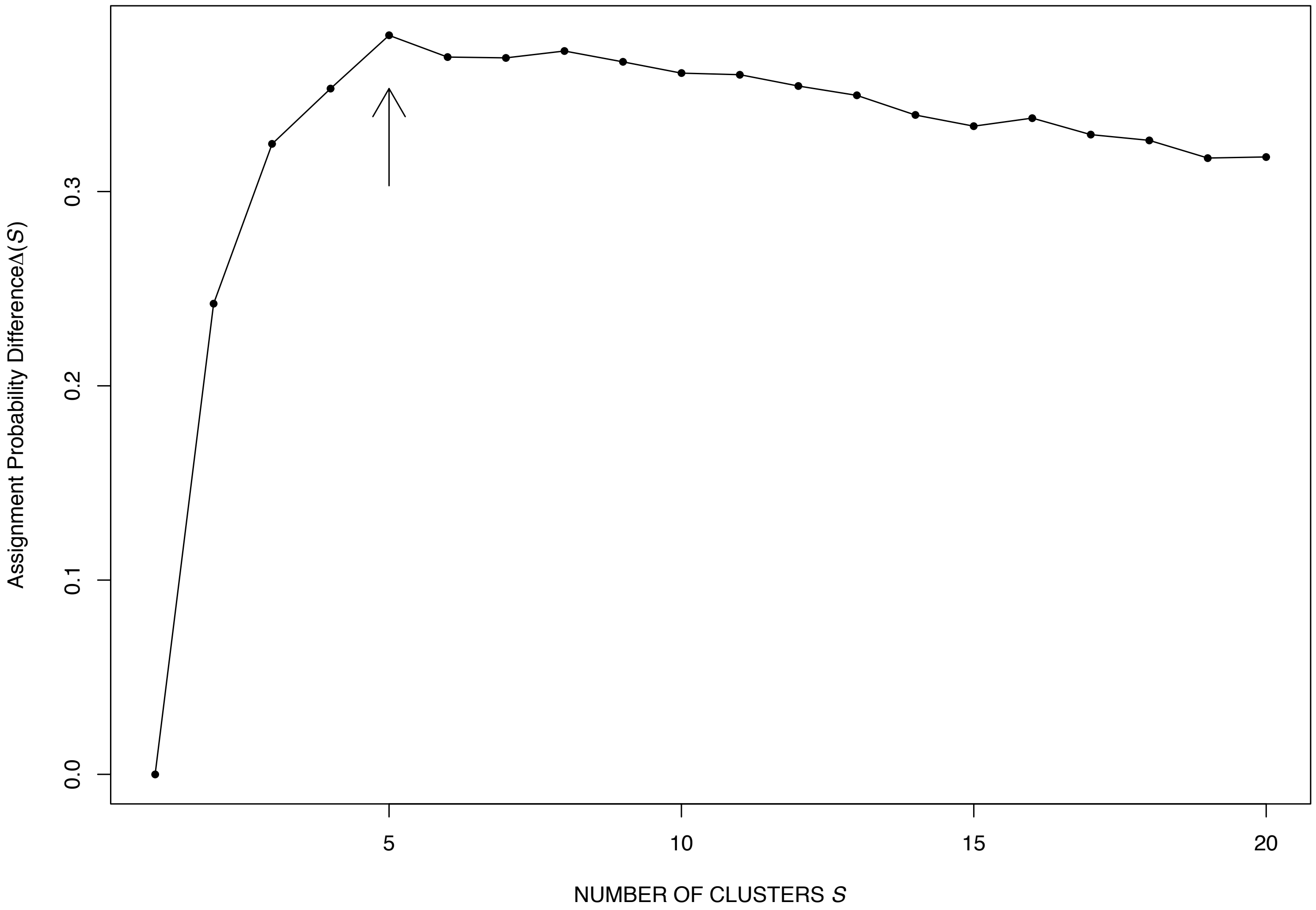

Figure S2 The cluster stability curves for (A) *Strongylocentrotus purpuratus* and (B) *Drosophila melanogaster*. The difference $\Delta(S) = B(S) - B_{random}(S)$ of the assignment probabilities is plotted against the number of cluster *S*.

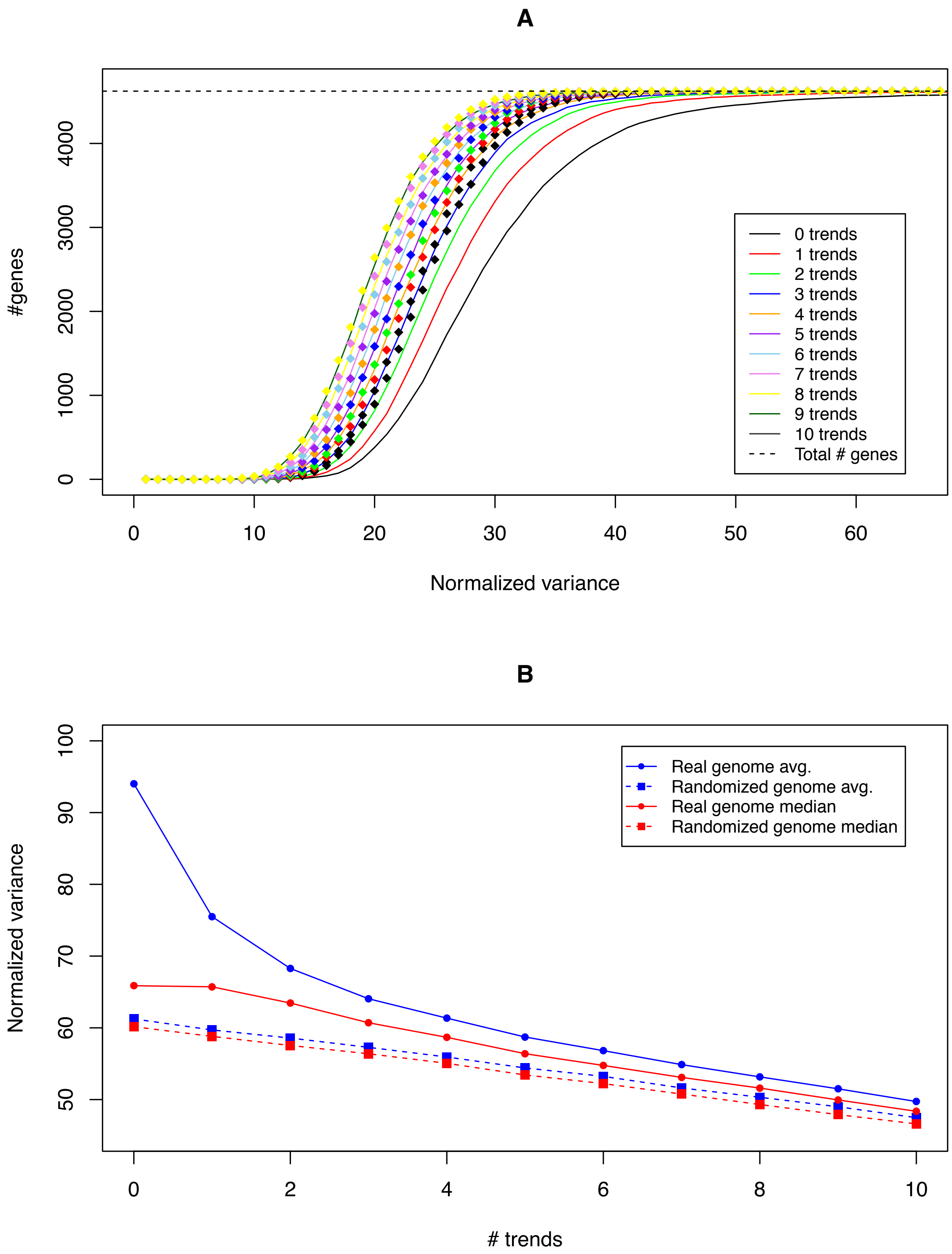

Figure S3 Normalized variance for SCUMBLE models with up to 10 trends in *S. purpuratus*. (A) Cumulative histogram of normalized variance for SCUMBLE models with up to 0-10 trends. (B) Normalized variance for SCUMBLE models with up to 10 trends.

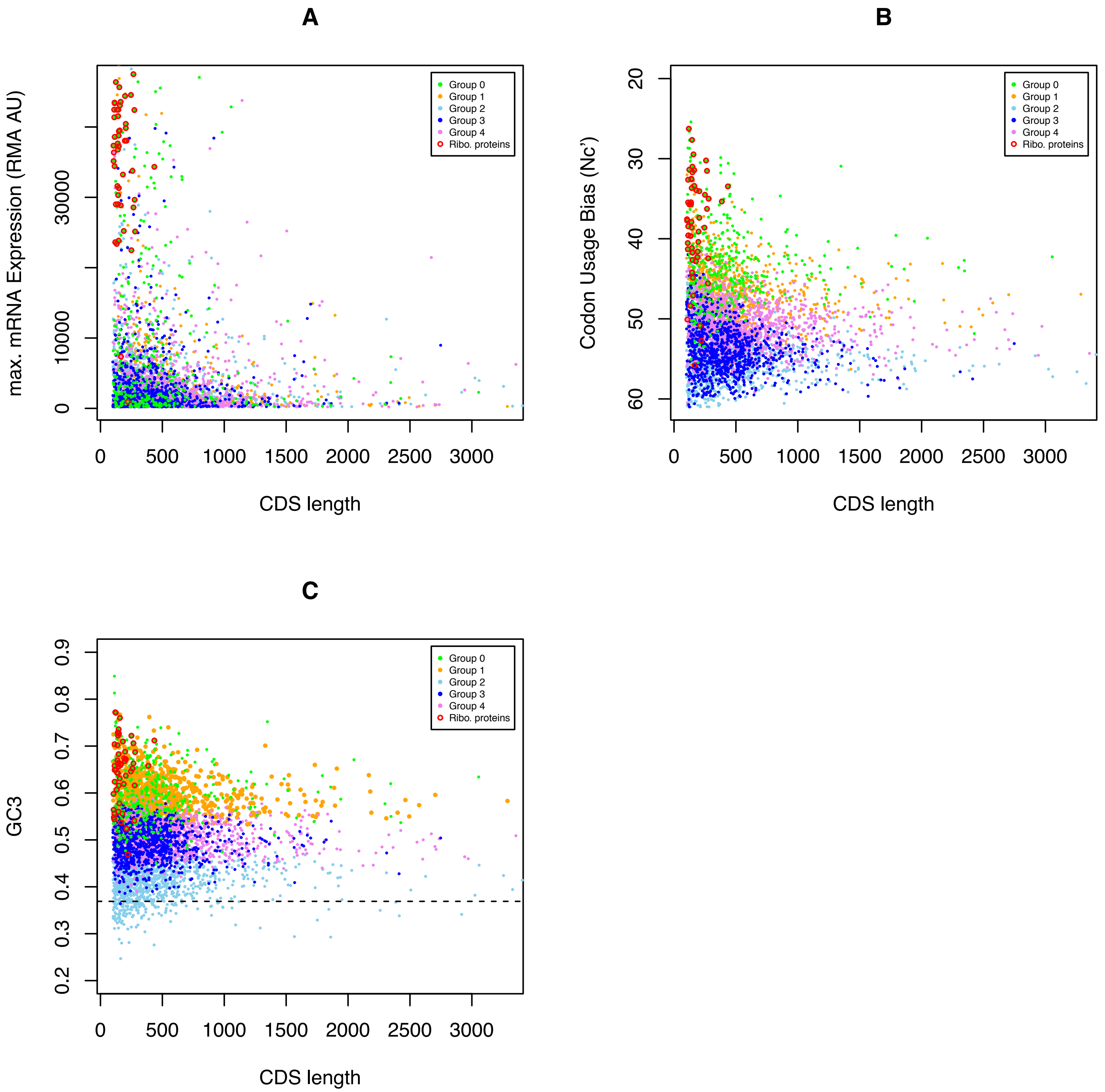

Figure S4 Coding sequence (CDS) length scatterplots. Coding sequence (CDS) length versus(A) mRNA expression level, (B) codon bias and (C) GC3 content (dashed horizontal line denotes genome-wide average GC content).

Files S1 and S2

Available for download at http://www.g3journal.org/lookup/suppl/doi:10.1534/g3.113.005769/-/DC1

File S1
D. melanogaster dataset

File S2
S. purpuratus dataset

Table S1 2x2 contingency table for a synonymous codon preference and mRNA secondary structure for a given gene.

	mRNA Secondary Structure	
Preference	N3 Stem	N3 Loop
Preferred	a	b
Unpreferred	c	d

Table S2 Counts of preferred N3 for each group by preference method.

Preferred N3	Synonymous Codon Usage Group 0	1	2	3	4	RF[a] All	Preference by Bias
A	1	1	7	7	4	5	0
C	12	13	1	6	6	5	15
G	4	4	3	2	4	3	3
T	1	0	7	3	4	5	0

[a] Relative frequency (RF) of the synonymous across all genes.

Table S3 Synonymous codon usage in *S. purpuratus*.

		Spearman Correlation		Synonymous Codon Usage Probabilities[b]					RF [c]
Amino Acid	Codon	ρ	*P-value* [a]	Group 0	Group1	Group2	Group3	Group4	All
Ala	GCA	0.3113	1.99E-104	0.1939	0.2493	**0.3615**	**0.2927**	0.3045	0.2955
	GCC [d]	**-0.4405**	**9.31E-219**	**0.4067**	**0.3507**	0.2133	0.2658	0.2836	0.2878
	GCG	0.0451	2.14E-03	0.0592	0.1507	0.0703	0.1592	0.0787	0.1026
	GCT	0.1324	1.55E-19	0.3402	0.2494	0.3549	0.2823	**0.3332**	**0.3141**
Arg	AGA	0.3020	4.55E-98	0.1602	0.2257	**0.3933**	**0.2573**	**0.3168**	**0.2943**
	AGG	-0.1786	1.90E-34	**0.2538**	**0.2697**	0.2624	0.2020	0.2932	0.2623
	CGA	0.1518	3.05E-25	0.1045	0.1259	0.1033	0.1752	0.0992	0.1192
	CGC	**-0.2134**	**8.89E-49**	0.1831	0.1417	0.0651	0.1116	0.0940	0.1066
	CGG	0.0619	2.56E-05	0.0450	0.1209	0.0647	0.1150	0.0717	0.0851
	CGT	-0.0214	1.46E-01	0.2534	0.1161	0.1112	0.1388	0.1252	0.1327
Asn	AAC	**-0.4523**	**5.70E-232**	**0.7150**	**0.6522**	0.4442	**0.5465**	**0.5552**	**0.5604**
	AAT	0.4535	2.42E-233	0.2850	0.3478	**0.5558**	0.4535	0.4448	0.4396
Asp	GAC	**-0.3373**	**2.06E-123**	**0.5290**	**0.5467**	0.3638	0.4595	0.4280	0.4472
	GAT	0.3383	3.48E-124	0.4710	0.4533	**0.6362**	**0.5405**	**0.5720**	**0.5528**
Cys	TGC	**-0.1914**	**2.13E-39**	**0.5552**	**0.5593**	0.3767	0.4645	0.4583	0.4682
	TGT	0.2081	2.18E-46	0.4448	0.4407	**0.6233**	**0.5355**	**0.5417**	**0.5318**
Gln	CAA	0.3820	1.73E-160	0.2794	0.3324	0.4594	0.4725	0.3551	0.3893
	CAG	**-0.3784**	**2.78E-157**	**0.7206**	**0.6676**	**0.5406**	**0.5275**	**0.6449**	**0.6107**
Glu	GAA	0.4160	6.46E-193	0.3345	0.3803	**0.5195**	**0.5115**	0.4175	0.4446
	GAG	**-0.4163**	**2.82E-193**	**0.6655**	**0.6197**	0.4805	0.4885	**0.5825**	**0.5554**
Gly	GGA	0.1497	1.44E-24	0.3236	**0.2784**	**0.3726**	**0.3114**	**0.3319**	**0.3269**
	GGC	**-0.2490**	**2.88E-66**	0.2534	0.2643	0.1683	0.2203	0.2048	0.2143
	GGG	0.0718	1.03E-06	0.0962	0.1983	0.1509	0.1871	0.1618	0.1654
	GGT	0.0521	3.99E-04	**0.3268**	0.2590	0.3082	0.2812	0.3015	0.2934
His	CAC	**-0.2282**	**1.10E-55**	**0.5230**	**0.5528**	0.3774	0.4562	0.4316	0.4534
	CAT	0.2416	2.24E-62	0.4770	0.4472	**0.6226**	**0.5438**	**0.5684**	**0.5466**
Ile	ATA	0.3847	6.16E-163	0.0658	0.1715	0.2556	0.2280	0.1916	0.1986
	ATC	**-0.5357**	**1.00E-223**	**0.7076**	**0.6101**	**0.4055**	**0.4772**	**0.5309**	**0.5220**
	ATT	0.3422	3.55E-127	0.2266	0.2185	0.3389	0.2948	0.2776	0.2793
Leu	TTA	0.4527	2.16E-232	0.0324	0.0741	0.1317	0.1209	0.0796	0.0942
	TTG	0.2742	1.67E-80	0.1136	0.1290	0.1641	0.1623	0.1417	0.1459
	CTA	0.2531	1.65E-68	0.0864	0.1046	0.1411	0.1241	0.1305	0.1235
	CTC	**-0.4612**	**2.87E-242**	**0.3035**	**0.2813**	0.1734	**0.2168**	0.2199	0.2267
	CTG	-0.3895	2.64E-167	0.2993	0.2663	0.1917	0.1953	**0.2503**	**0.2339**
	CTT	0.2080	2.28E-46	0.1649	0.1447	**0.1979**	0.1806	0.1780	0.1757
Lys	AAA	0.4662	2.95E-248	0.2195	0.3375	0.4490	0.4707	0.3577	0.3829
	AAG	**-0.4665**	**1.36E-248**	**0.7805**	**0.6625**	**0.5510**	**0.5293**	**0.6423**	**0.6171**
Phe	TTC	**-0.3821**	**1.43E-160**	**0.6961**	**0.6610**	0.4986	**0.5662**	**0.5772**	**0.5841**
	TTT	0.3813	7.54E-160	0.3039	0.3390	**0.5014**	0.4338	0.4228	0.4159
Pro	CCA	0.1854	5.08E-37	**0.3162**	0.2661	**0.4055**	**0.3238**	**0.3471**	**0.3379**
	CCC	**-0.3173**	**1.19E-108**	0.2907	**0.2864**	0.1607	0.2120	0.2215	0.2236

	CCG	-0.0108	4.64E-01	0.1078	0.1889	0.0880	0.1927	0.1019	0.1330
	CCT	0.1568	7.61E-27	0.2853	0.2585	0.3458	0.2715	0.3296	0.3055
Ser	AGC	-0.2299	1.61E-56	0.2111	**0.2121**	0.1497	0.1654	0.1842	0.1796
	AGT	0.2037	1.68E-44	0.1392	0.1558	0.2080	0.1677	0.1900	0.1805
	TCA	0.2309	5.12E-57	0.1460	0.1654	**0.2525**	**0.2077**	**0.2079**	**0.2065**
	TCC	**-0.2789**	**2.19E-83**	**0.2333**	0.1971	0.1258	0.1506	0.1681	0.1645
	TCG	-0.0224	1.27E-01	0.0813	0.1366	0.0585	0.1463	0.0684	0.0940
	TCT	0.1907	3.94E-39	0.1890	0.1330	0.2055	0.1623	0.1815	0.1750
Thr	ACA	0.2837	2.66E-86	0.2151	0.2550	**0.3990**	**0.3125**	0.3235	**0.3171**
	ACC	**-0.4199**	**6.96E-197**	**0.4664**	**0.3608**	0.2339	0.2690	**0.3268**	0.3123
	ACG	-0.0391	7.81E-03	0.1168	0.2267	0.1063	0.2162	0.1308	0.1584
	ACT	0.2770	3.53E-82	0.2016	0.1575	0.2608	0.2023	0.2189	0.2122
Tyr	TAC	**-0.3811**	**1.12E-159**	**0.7296**	**0.6901**	0.4892	**0.5711**	**0.5892**	**0.5955**
	TAT	0.3867	9.07E-165	0.2704	0.3099	**0.5108**	0.4289	0.4108	0.4045
Val	GTA	0.3086	1.48E-102	0.1357	0.1431	0.2329	0.1878	0.1803	0.1832
	GTC	**-0.3935**	**4.42E-171**	**0.4005**	**0.3736**	0.2378	**0.3045**	**0.3047**	**0.3099**
	GTG	-0.0946	1.17E-10	0.2758	0.3089	**0.2695**	0.2685	0.3006	0.2873
	GTT	0.3459	4.56E-130	0.1880	0.1744	0.2598	0.2392	0.2143	0.2196

[a] The most significant negative correlation are highlighted in bold.

[b] The probability a given gene in the group will use the synonymous codon is taken as the relative frequency of the codon in its family for all genes in the group. The highest frequency of a codon family is in bold.

[c] The relative frequency of the synonymous codon for the amino acid for all genes. The highest frequency of a codon family is in bold.

Table S4 The strongest and most significant Spearman correlation coefficients for each of the first three SCUMBLE offsets for a SCUMBLE model with 4 trends.

Group	β_1		β_2		β_3	
0	GC3, 0.8443	***	72h[a], 0.4053	***	CT3, 0.5120	***
1	GC3, 0.8545	***	CT3, 0.1748	***	CT3, 0.4917	***
2	GC3, 0.7724	***	GT3, 0.3189	***	CT3, 0.4735	***
3	GC3, 0.8108	***	CT3, 0.3315	***	CT3, 0.4923	***
4	GC3, 0.7089	***	CT3, 0.3015	***	CT3, 0.5142	***
All	GC3, 0.9961	***	72h, 0.3277	***	CT3, 0.4450	***

*** *P-value*$< 1x10^{-10}$

* *P-value*< 0.001

[a] Expression value at 72h after fertilization.

Table S5 Correlations between codon bias (Nc), regional GC content and rates of protein evolution in *Strongylocentrotus purpuratus*.

		Spearman's Correlation Coefficient for each group [a]					
		Group 0	Group 1	Group 2	Group 3	Group 4	All
Codon Bias							
Nc	GC3	-0.5524***	-0.4653***	0.2628***	0.1435***	-0.0202	-0.054*
Nc	GC_{cds}	-0.442***	-0.2217***	0.2394***	0.0896	0.0255	-0.0219
Nc	GC_i[b]	-0.0431	0.0196	0.1365*	0.0365	0.0098	0.0967*
Nc	GC_f	-0.002	-0.0274	0.102*	-0.0314	0.0125	0.0555*
Rate Comparisons[c]							
Nc	*dS*	-0.0237	-0.1361*	-0.0031	-0.0083	-0.019	-0.0519
Nc	*dN*	0.1401	0.1507*	0.0644	0.0955	0.1111	0.1293***
Nc	*dN/dS*	0.1541	0.1967*	0.0557	0.0982	0.1076	0.1407***

* Significance at $P < 0.001$

*** Significance at $P < 10^{-10}$

[a] Number of genes in each group: All (4623), Cluster 0 (396), Cluster 1 (861), Cluster 2 (1154), Cluster 3 (912), Cluster 4 (1300)

[b] Number of genes with introns for each group: All (4389), Cluster 0 (368), Cluster 1 (814), Cluster 2 (1113), Cluster 3 (826), Cluster 4 (1268)

[c] Number of genes with comparative data for each group: All (2954), Cluster 0 (225), Cluster 1 (593), Cluster 2 (744), Cluster 3 (563), Cluster 4 (829)

Table S6. Significantly Over-Enriched Gene Ontology (GO) codes for *S. purpuratus* gene groups.

Group	GO_ID	GO Term	Domain[a]	FDR[b]	*P-value*
C0	GO:0005198	structural molecule activity	F	0	0
C0	GO:0005840	ribosome	C	3.67E-36	5.20E-38
C0	GO:0030529	ribonucleoprotein complex	C	3.67E-36	5.20E-38
C0	GO:0006412	translation	P	2.05E-33	3.86E-35
C0	GO:0032991	macromolecular complex	C	3.14E-32	7.41E-34
C0	GO:0005829	cytosol	C	7.27E-25	2.06E-26
C0	GO:0043228	non-membrane-bounded organelle	C	2.04E-20	7.71E-22
C0	GO:0043232	intracellular non-membrane-bounded organelle	C	2.04E-20	7.71E-22
C0	GO:0003723	RNA binding	F	4.59E-14	1.95E-15
C0	GO:0005737	cytoplasm	C	1.91E-13	8.99E-15
C0	GO:0000166	nucleotide binding	F	7.13E-12	3.70E-13
C0	GO:0030312	external encapsulating structure	C	1.61E-11	9.12E-13
C0	GO:0044444	cytoplasmic part	C	3.40E-11	2.17E-12
C0	GO:0005929	cilium	C	3.40E-11	2.41E-12
C0	GO:0042995	cell projection	C	3.40E-11	2.41E-12
C0	GO:0005811	lipid particle	C	7.86E-11	5.93E-12
C0	GO:0005618	cell wall	C	1.47E-10	1.18E-11
C0	GO:0043234	protein complex	C	1.63E-10	1.38E-11
C0	GO:0009536	plastid	C	1.02E-09	9.13E-11
C0	GO:0005856	cytoskeleton	C	6.87E-09	6.48E-10
C0	GO:0044424	intracellular part	C	1.63E-08	1.61E-09
C0	GO:0043229	intracellular organelle	C	2.64E-08	2.74E-09
C0	GO:0040007	growth	P	2.06E-07	2.23E-08
C0	GO:0009987	cellular process	P	2.47E-07	2.80E-08
C0	GO:0044267	cellular protein metabolic process	P	3.95E-07	4.66E-08
C0	GO:0005622	intracellular	C	5.87E-07	7.20E-08
C0	GO:0019538	protein metabolic process	P	1.28E-06	1.63E-07

C0	GO:0006996	organelle organization	P	1.64E-06	2.16E-07
C0	GO:0010467	gene expression	P	2.59E-06	3.54E-07
C0	GO:0044464	cell part	C	7.47E-06	1.06E-06
C0	GO:0044249	cellular biosynthetic process	P	1.59E-05	2.56E-06
C0	GO:0009059	macromolecule biosynthetic process	P	1.59E-05	2.56E-06
C0	GO:0034645	cellular macromolecule biosynthetic process	P	1.59E-05	2.56E-06
C0	GO:0005515	protein binding	F	1.59E-05	2.62E-06
C0	GO:0043226	organelle	C	1.59E-05	2.62E-06
C0	GO:0016043	cellular component organization	P	2.51E-05	4.63E-06
C0	GO:0003774	motor activity	F	2.51E-05	4.86E-06
C0	GO:0016818	hydrolase activity, acting on acid anhydrides, in phosphorus-containing anhydrides	F	2.51E-05	4.86E-06
C0	GO:0016817	hydrolase activity, acting on acid anhydrides	F	2.51E-05	4.86E-06
C0	GO:0016462	pyrophosphatase activity	F	2.51E-05	4.86E-06
C0	GO:0017111	nucleoside-triphosphatase activity	F	2.51E-05	4.86E-06
C0	GO:0007010	cytoskeleton organization	P	3.74E-05	7.42E-06
C0	GO:0003676	nucleic acid binding	F	9.70E-05	1.97E-05
C0	GO:0009055	electron carrier activity	F	0.00075772	0.000157263
C0	GO:0009790	embryonic development	P	0.000759207	0.000161152
C0	GO:0044422	organelle part	C	0.000948809	0.000210349
C0	GO:0044446	intracellular organelle part	C	0.000948809	0.000210349
C0	GO:0044237	cellular metabolic process	P	0.00152677	0.000345684
C0	GO:0045182	translation regulator activity	F	0.00187651	0.000433721
C0	GO:0030313	cell envelope	C	0.0020718	0.000488631
C0	GO:0007049	cell cycle	P	0.00259108	0.000623326
C0	GO:0065008	regulation of biological quality	P	0.00356715	0.000874962
C0	GO:0005730	nucleolus	C	0.0039766	0.00099415
C0	GO:0006091	generation of precursor metabolites and energy	P	0.00424169	0.00108043
C0	GO:0000003	reproduction	P	0.00441887	0.0011464
C0	GO:0005488	binding	F	0.00627651	0.00165794

C0	GO:0043170	macromolecule metabolic process	P	0.00630195	0.00169439
C0	GO:0005623	cell	C	0.00859926	0.00235263
C0	GO:0044428	nuclear part	C	0.00925341	0.00257524
C0	GO:0016209	antioxidant activity	F	0.0111415	0.00315326
C0	GO:0005509	calcium ion binding	F	0.0138875	0.00399592
C0	GO:0043167	ion binding	F	0.0154194	0.00465491
C0	GO:0043169	cation binding	F	0.0154194	0.00465491
C0	GO:0046872	metal ion binding	F	0.0154194	0.00465491
C0	GO:0005102	receptor binding	F	0.020271	0.00621516
C0	GO:0019725	cellular homeostasis	P	0.0210874	0.00666442
C0	GO:0042592	homeostatic process	P	0.0210874	0.00666442
C0	GO:0031974	membrane-enclosed lumen	C	0.021475	0.00719209
C0	GO:0043233	organelle lumen	C	0.021475	0.00719209
C0	GO:0070013	intracellular organelle lumen	C	0.021475	0.00719209
C0	GO:0031981	nuclear lumen	C	0.021475	0.00719209
C0	GO:0005773	vacuole	C	0.025567	0.00868312
C0	GO:0005764	lysosome	C	0.0260626	0.00909732
C0	GO:0000323	lytic vacuole	C	0.0260626	0.00909732
C0	GO:0009058	biosynthetic process	P	0.0261996	0.00926873
C0	GO:0005634	nucleus	C	0.0275011	0.0098589
C0	GO:0005815	microtubule organizing center	C	0.0289569	0.0107905
C0	GO:0044430	cytoskeletal part	C	0.0289569	0.0107905
C0	GO:0015630	microtubule cytoskeleton	C	0.0289569	0.0107905
C0	GO:0044260	cellular macromolecule metabolic process	P	0.0289666	0.0109308
C0	GO:0008219	cell death	P	0.0320537	0.0122469
C0	GO:0032502	developmental process	P	0.0342324	0.0132408
C0	GO:0030246	carbohydrate binding	F	0.0364777	0.0142814
C0	GO:0016265	death	P	0.03887	0.0154444
C0	GO:0005886	plasma membrane	C	0.03887	0.015768

C0	GO:0016020	membrane	C	0.03887	0.015768
C0	GO:0016049	cell growth	P	0.0447576	0.0190009
C0	GO:0090066	regulation of anatomical structure size	P	0.0447576	0.0190009
C0	GO:0032535	regulation of cellular component size	P	0.0447576	0.0190009
C0	GO:0008361	regulation of cell size	P	0.0447576	0.0190009
C1	GO:0030528	transcription regulator activity	F	3.04E-18	2.13E-20
C1	GO:0006350	transcription	P	3.04E-18	2.86E-20
C1	GO:0090304	nucleic acid metabolic process	P	8.06E-14	2.66E-15
C1	GO:0003700	transcription factor activity	F	3.64E-12	1.37E-13
C1	GO:0006139	nucleobase, nucleoside, nucleotide and nucleic acid metabolic process	P	1.48E-10	7.66E-12
C1	GO:0006807	nitrogen compound metabolic process	P	1.48E-10	7.66E-12
C1	GO:0034641	cellular nitrogen compound metabolic process	P	1.48E-10	7.66E-12
C1	GO:0008283	cell proliferation	P	5.31E-07	3.26E-08
C1	GO:0023052	signaling	P	1.44E-06	1.06E-07
C1	GO:0009653	anatomical structure morphogenesis	P	2.40E-06	2.04E-07
C1	GO:0048856	anatomical structure development	P	2.40E-06	2.04E-07
C1	GO:0007165	signal transduction	P	5.54E-06	5.75E-07
C1	GO:0023046	signaling process	P	5.54E-06	5.75E-07
C1	GO:0050794	regulation of cellular process	P	5.54E-06	5.75E-07
C1	GO:0023060	signal transmission	P	5.54E-06	5.75E-07
C1	GO:0030154	cell differentiation	P	5.63E-06	6.37E-07
C1	GO:0048869	cellular developmental process	P	5.63E-06	6.37E-07
C1	GO:0003677	DNA binding	F	2.11E-05	2.49E-06
C1	GO:0005215	transporter activity	F	5.54E-05	6.80E-06
C1	GO:0007267	cell-cell signaling	P	0.000245924	3.25E-05
C1	GO:0007610	behavior	P	0.000478496	6.55E-05
C1	GO:0051179	localization	P	0.000484876	7.45E-05
C1	GO:0006810	transport	P	0.000484876	7.45E-05
C1	GO:0051234	establishment of localization	P	0.000484876	7.45E-05

C1	GO:0032501	multicellular organismal process	P	0.000484876	7.55E-05
C1	GO:0007275	multicellular organismal development	P	0.00049517	7.94E-05
C1	GO:0050789	regulation of biological process	P	0.00277685	0.000484638
C1	GO:0006811	ion transport	P	0.00373494	0.000687088
C1	GO:0022857	transmembrane transporter activity	F	0.00439264	0.0008288
C1	GO:0065007	biological regulation	P	0.00448243	0.000866886
C1	GO:0043227	membrane-bounded organelle	C	0.00505752	0.00112408
C1	GO:0043231	intracellular membrane-bounded organelle	C	0.00505752	0.00112408
C1	GO:0005216	ion channel activity	F	0.00505752	0.00119281
C1	GO:0022892	substrate-specific transporter activity	F	0.00505752	0.00119281
C1	GO:0022803	passive transmembrane transporter activity	F	0.00505752	0.00119281
C1	GO:0022891	substrate-specific transmembrane transporter activity	F	0.00505752	0.00119281
C1	GO:0015267	channel activity	F	0.00505752	0.00119281
C1	GO:0022838	substrate-specific channel activity	F	0.00505752	0.00119281
C1	GO:0015075	ion transmembrane transporter activity	F	0.00505752	0.00119281
C1	GO:0007154	cell communication	P	0.0073416	0.00176614
C1	GO:0003682	chromatin binding	F	0.00852481	0.00209099
C1	GO:0006464	protein modification process	P	0.0140444	0.00384233
C1	GO:0043412	macromolecule modification	P	0.0140444	0.00384233
C1	GO:0003779	actin binding	F	0.0234774	0.00686602
C1	GO:0008289	lipid binding	F	0.0321267	0.00998075
C1	GO:0030234	enzyme regulator activity	F	0.0411592	0.0141727
C2	GO:0005739	mitochondrion	C	0.00106027	2.50E-05
C2	GO:0003824	catalytic activity	F	0.00159266	6.14E-05
C2	GO:0015031	protein transport	P	0.00159266	8.70E-05
C2	GO:0033036	macromolecule localization	P	0.00159266	8.70E-05
C2	GO:0008104	protein localization	P	0.00159266	8.70E-05
C2	GO:0045184	establishment of protein localization	P	0.00159266	8.70E-05
C2	GO:0005794	Golgi apparatus	C	0.00159266	8.79E-05

C2	GO:0006259	DNA metabolic process	P	0.00280385	0.000198386
C2	GO:0016788	hydrolase activity, acting on ester bonds	F	0.026659	0.00238925
C3	GO:0009719	response to endogenous stimulus	P	0.000191016	4.51E-06
C3	GO:0005975	carbohydrate metabolic process	P	0.00102028	3.85E-05
C3	GO:0044421	extracellular region part	C	0.00651626	0.000430319
C3	GO:0005615	extracellular space	C	0.00995884	0.000845561
C3	GO:0016740	transferase activity	F	0.0182658	0.00172319
C3	GO:0005768	endosome	C	0.0316677	0.00388378
C3	GO:0044238	primary metabolic process	P	0.0337436	0.00429753
C4	GO:0008152	metabolic process	P	7.88E-06	1.08E-06
C4	GO:0004672	protein kinase activity	F	8.30E-05	1.46E-05
C4	GO:0016773	phosphotransferase activity, alcohol group as acceptor	F	8.30E-05	1.46E-05
C4	GO:0016772	transferase activity, transferring phosphorus-containing groups	F	8.30E-05	1.53E-05
C4	GO:0016301	kinase activity	F	8.30E-05	1.53E-05
C4	GO:0009056	catabolic process	P	0.000764796	0.000151516
C4	GO:0016787	hydrolase activity	F	0.00104996	0.000232775
C4	GO:0016032	viral reproduction	P	0.0254601	0.00744587

[a]GO domain: cellular component (‘C’), biological process (‘B’) and molecular function (‘F’)
[b] False Discovery Rate
