# Supplementary figures and images for: Genome-Wide Patterns of Codon Bias Are Shaped by Natural Selection in the Purple Sea Urchin, Strongylocentrotus purpuratus
Source: G3 (Bethesda). 2013 Jul 1;3(7):1069–83. doi: 10.1534/g3.113.005769 (PMC3704236; doi:10.1534/g3.113.005769)

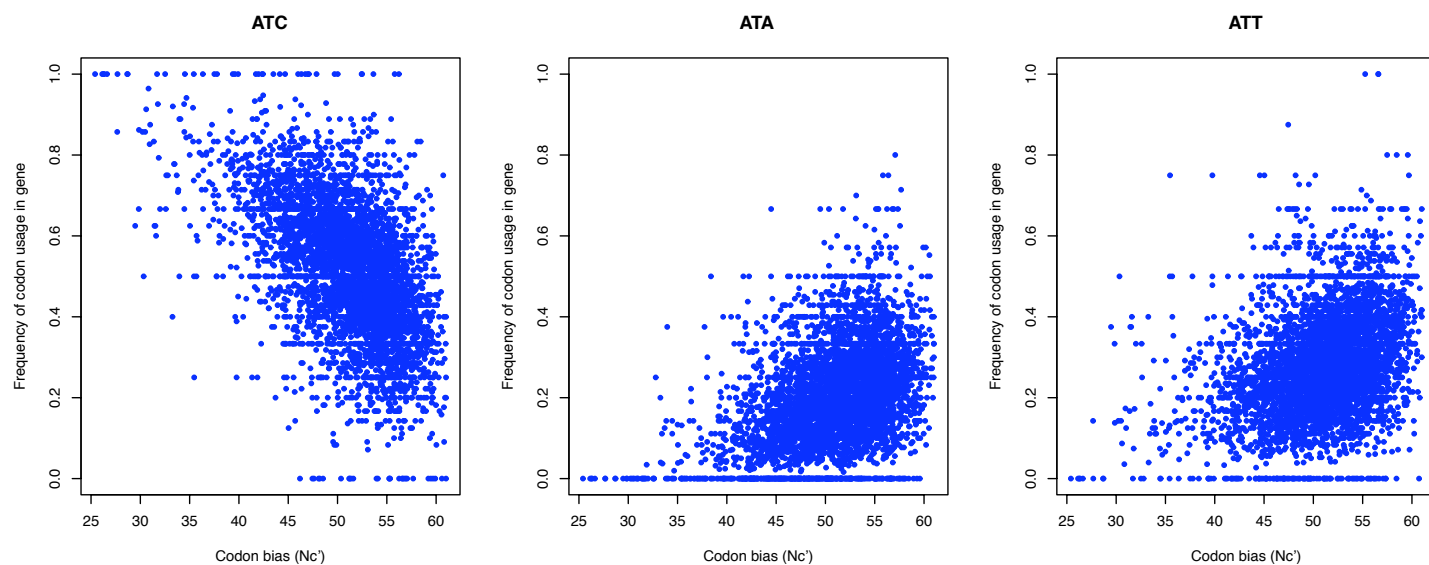

**Figure S1** Genome-wide preferred codon usage for Ile.

Supplement: Supporting Information [file supp_g3.113.005769_FigureS1.pdf]
