# Supplementary material for: Genome-Wide Patterns of Codon Bias Are Shaped by Natural Selection in the Purple Sea Urchin, Strongylocentrotus purpuratus
Source: G3 (Bethesda). 2013 Jul 1;3(7):1069–83. doi: 10.1534/g3.113.005769 (PMC3704236; doi:10.1534/g3.113.005769)
Supplement: Supporting Information [file supp_g3.113.005769_FigureS2.pdf]

A

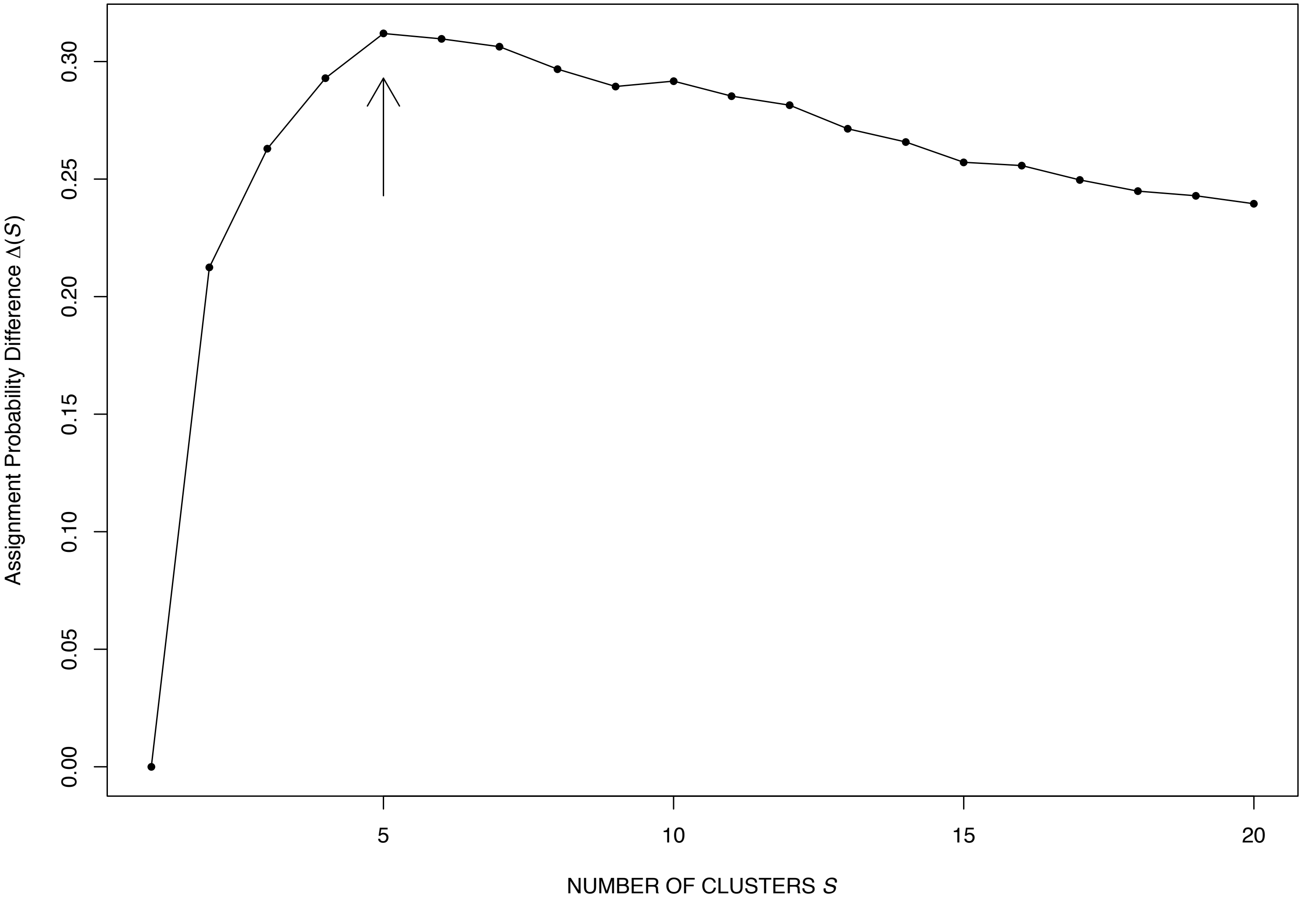

Assignment Probability Difference Δ(S)
0.00
0.05
0.10
0.15
0.20
0.25
0.30
5
10
15
20
NUMBER OF CLUSTERS S

B

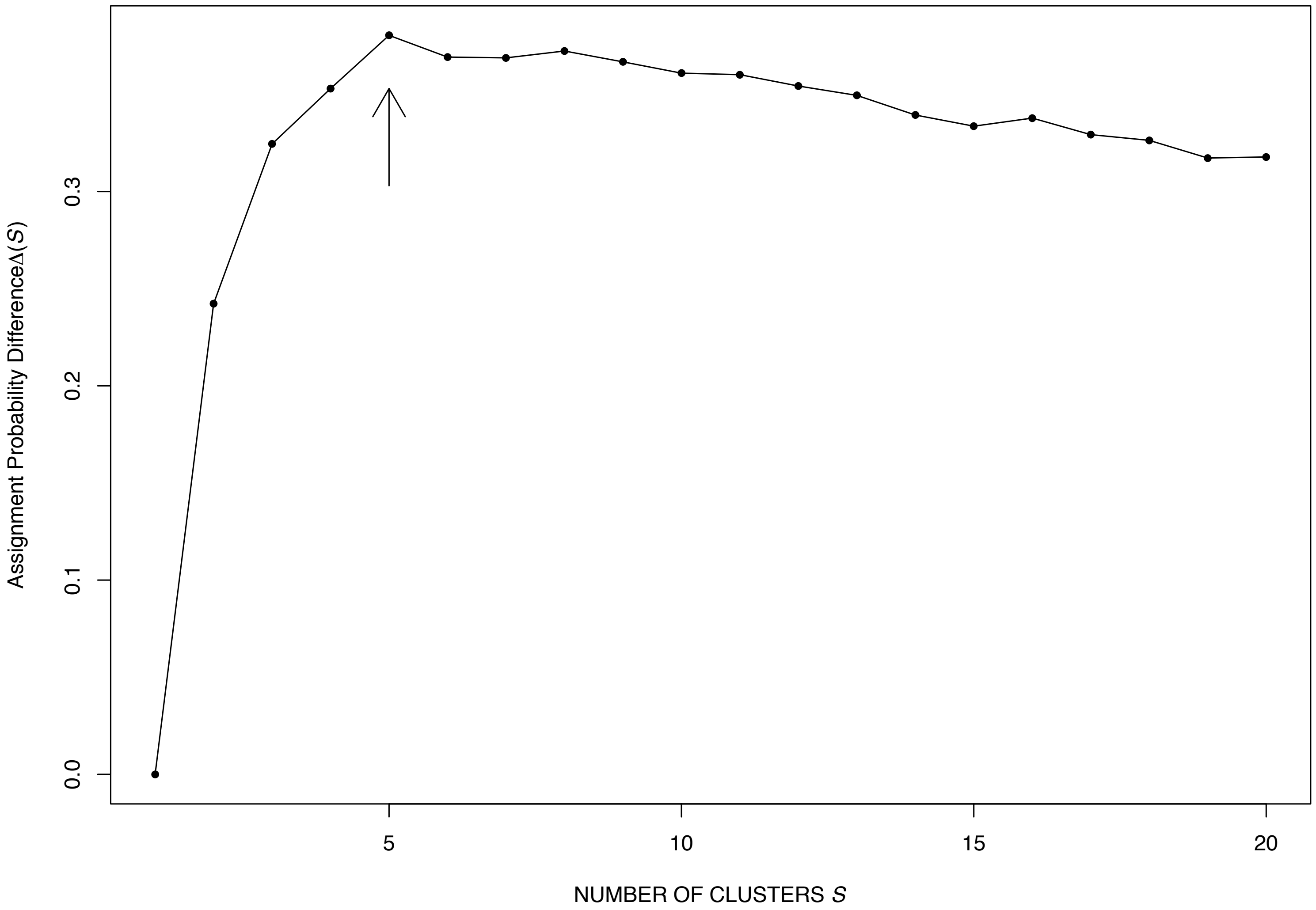

Figure S2 The cluster stability curves for (A) *Strongylocentrotus purpuratus* and (B) *Drosophila melanogaster*. The difference $\Delta(S) = B(S) - B_{random}(S)$ of the assignment probabilities is plotted against the number of cluster *S*.
