# Supplementary material for: Genome-Wide Patterns of Codon Bias Are Shaped by Natural Selection in the Purple Sea Urchin, Strongylocentrotus purpuratus
Source: G3 (Bethesda). 2013 Jul 1;3(7):1069–83. doi: 10.1534/g3.113.005769 (PMC3704236; doi:10.1534/g3.113.005769)
Supplement: Supporting Information [file supp_g3.113.005769_FigureS3.pdf]

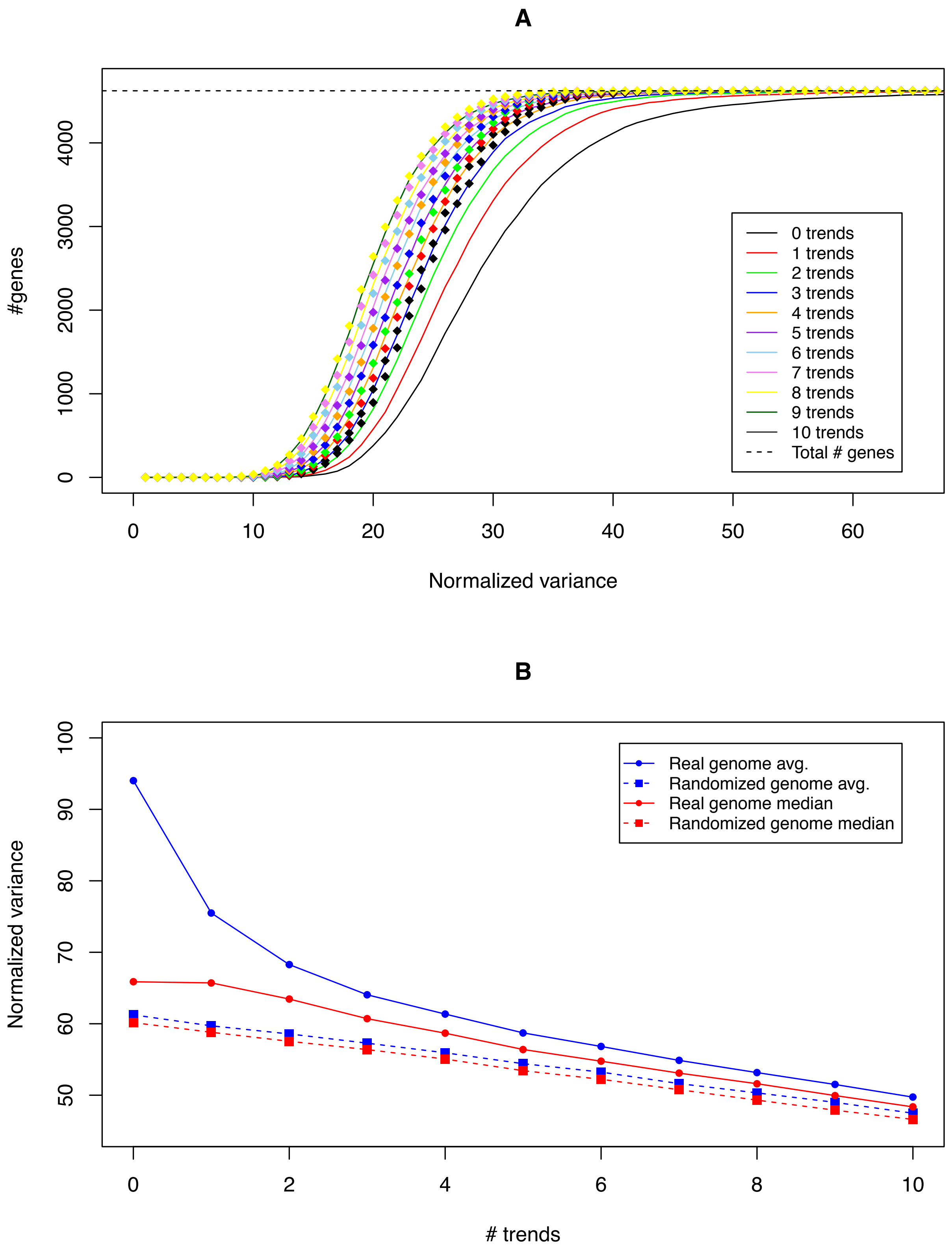

Figure S3 Normalized variance for SCUMBLE models with up to 10 trends in *S. purpuratus*. (A) Cumulative histogram of normalized variance for SCUMBLE models with up to 0-10 trends. (B) Normalized variance for SCUMBLE models with up to 10 trends.
