# Supplementary material for: Genome-Wide Patterns of Codon Bias Are Shaped by Natural Selection in the Purple Sea Urchin, Strongylocentrotus purpuratus
Source: G3 (Bethesda). 2013 Jul 1;3(7):1069–83. doi: 10.1534/g3.113.005769 (PMC3704236; doi:10.1534/g3.113.005769)
Supplement: Supporting Information [file supp_g3.113.005769_FigureS4.pdf]

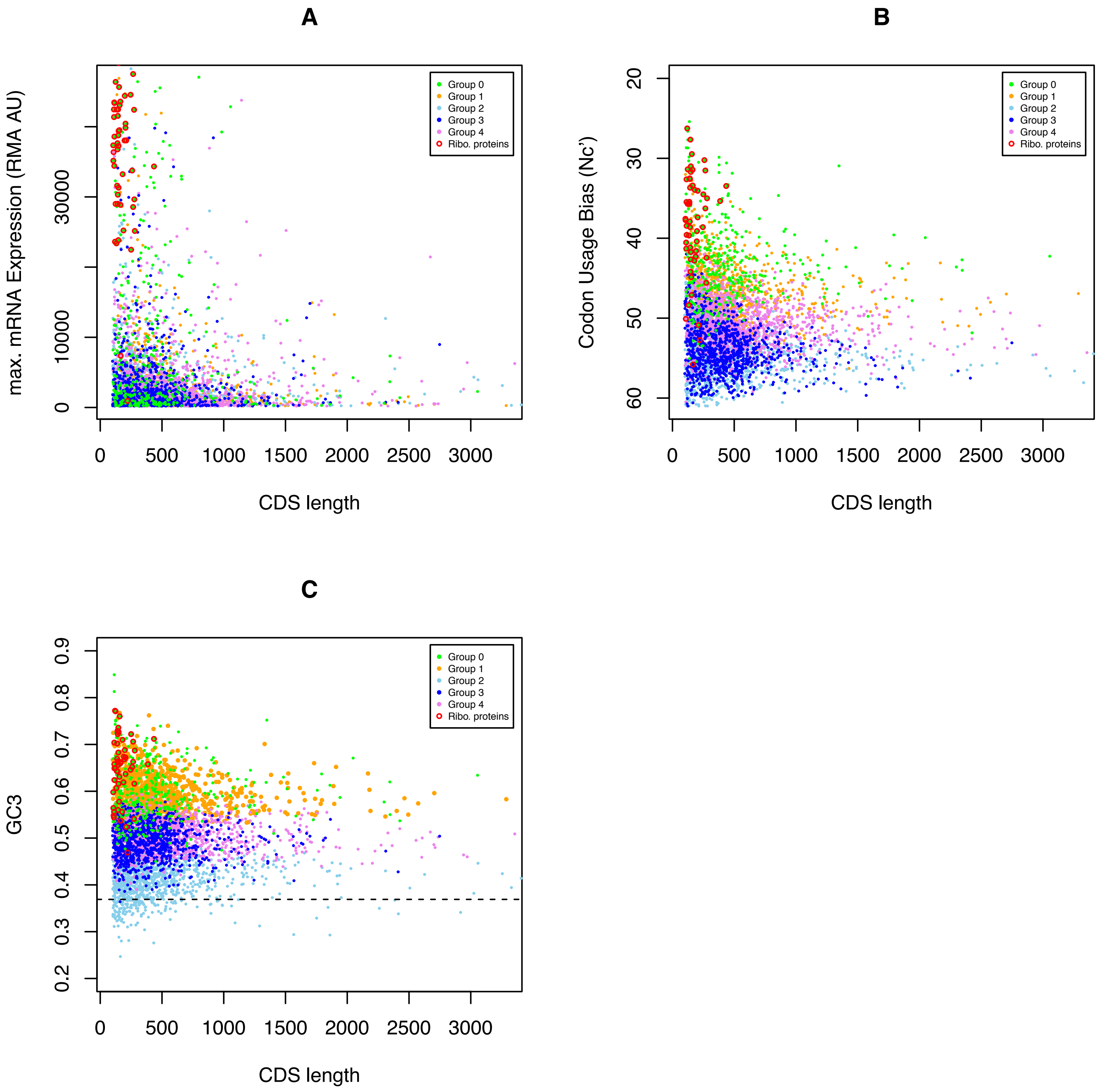

Figure S4 Coding sequence (CDS) length scatterplots. Coding sequence (CDS) length versus(A) mRNA expression level, (B) codon bias and (C) GC3 content (dashed horizontal line denotes genome-wide average GC content).
