# Supplementary material for: Genome-Wide Patterns of Codon Bias Are Shaped by Natural Selection in the Purple Sea Urchin, Strongylocentrotus purpuratus
Source: G3 (Bethesda). 2013 Jul 1;3(7):1069–83. doi: 10.1534/g3.113.005769 (PMC3704236; doi:10.1534/g3.113.005769)
Supplement: Supporting Information [file supp_g3.113.005769_TableS1.pdf]

Table S1 2x2 contingency table for a synonymous codon preference and mRNA secondary structure for a given gene.

	mRNA Secondary Structure	
Preference	N3 Stem	N3 Loop
Preferred	a	b
Unpreferred	c	d
