# Supplementary material for: Genome-Wide Patterns of Codon Bias Are Shaped by Natural Selection in the Purple Sea Urchin, Strongylocentrotus purpuratus
Source: G3 (Bethesda). 2013 Jul 1;3(7):1069–83. doi: 10.1534/g3.113.005769 (PMC3704236; doi:10.1534/g3.113.005769)
Supplement: Supporting Information [file supp_g3.113.005769_TableS2.pdf]

Table S2 Counts of preferred N3 for each group by preference method.

Preferred	Synonymous Codon Usage Group					RF[a]	Preference
N3	0	1	2	3	4	All	by Bias
A	1	1	7	7	4	5	0
C	12	13	1	6	6	5	15
G	4	4	3	2	4	3	3
T	1	0	7	3	4	5	0

[a] Relative frequency (RF) of the synonymous across all genes.
