# Supplementary material for: Genome-Wide Patterns of Codon Bias Are Shaped by Natural Selection in the Purple Sea Urchin, Strongylocentrotus purpuratus
Source: G3 (Bethesda). 2013 Jul 1;3(7):1069–83. doi: 10.1534/g3.113.005769 (PMC3704236; doi:10.1534/g3.113.005769)
Supplement: Supporting Information [file supp_g3.113.005769_TableS3.pdf]

Table S3 Synonymous codon usage in *S. purpuratus*.

		Spearman Correlation		Synonymous Codon Usage Probabilities[b]					RF [c]
Amino Acid	Codon	ρ	*P-value* [a]	Group 0	Group1	Group2	Group3	Group4	All
Ala	GCA	0.3113	1.99E-104	0.1939	0.2493	**0.3615**	**0.2927**	0.3045	0.2955
	GCC [d]	**-0.4405**	**9.31E-219**	**0.4067**	**0.3507**	0.2133	0.2658	0.2836	0.2878
	GCG	0.0451	2.14E-03	0.0592	0.1507	0.0703	0.1592	0.0787	0.1026
	GCT	0.1324	1.55E-19	0.3402	0.2494	0.3549	0.2823	**0.3332**	**0.3141**
Arg	AGA	0.3020	4.55E-98	0.1602	0.2257	**0.3933**	**0.2573**	**0.3168**	**0.2943**
	AGG	-0.1786	1.90E-34	**0.2538**	**0.2697**	0.2624	0.2020	0.2932	0.2623
	CGA	0.1518	3.05E-25	0.1045	0.1259	0.1033	0.1752	0.0992	0.1192
	CGC	**-0.2134**	**8.89E-49**	0.1831	0.1417	0.0651	0.1116	0.0940	0.1066
	CGG	0.0619	2.56E-05	0.0450	0.1209	0.0647	0.1150	0.0717	0.0851
	CGT	-0.0214	1.46E-01	0.2534	0.1161	0.1112	0.1388	0.1252	0.1327
Asn	AAC	**-0.4523**	**5.70E-232**	**0.7150**	**0.6522**	0.4442	**0.5465**	**0.5552**	**0.5604**
	AAT	0.4535	2.42E-233	0.2850	0.3478	**0.5558**	0.4535	0.4448	0.4396
Asp	GAC	**-0.3373**	**2.06E-123**	**0.5290**	**0.5467**	0.3638	0.4595	0.4280	0.4472
	GAT	0.3383	3.48E-124	0.4710	0.4533	**0.6362**	**0.5405**	**0.5720**	**0.5528**
Cys	TGC	**-0.1914**	**2.13E-39**	**0.5552**	**0.5593**	0.3767	0.4645	0.4583	0.4682
	TGT	0.2081	2.18E-46	0.4448	0.4407	**0.6233**	**0.5355**	**0.5417**	**0.5318**
Gln	CAA	0.3820	1.73E-160	0.2794	0.3324	0.4594	0.4725	0.3551	0.3893
	CAG	**-0.3784**	**2.78E-157**	**0.7206**	**0.6676**	**0.5406**	**0.5275**	**0.6449**	**0.6107**
Glu	GAA	0.4160	6.46E-193	0.3345	0.3803	**0.5195**	**0.5115**	0.4175	0.4446
	GAG	**-0.4163**	**2.82E-193**	**0.6655**	**0.6197**	0.4805	0.4885	**0.5825**	**0.5554**
Gly	GGA	0.1497	1.44E-24	0.3236	**0.2784**	**0.3726**	**0.3114**	**0.3319**	**0.3269**
	GGC	**-0.2490**	**2.88E-66**	0.2534	0.2643	0.1683	0.2203	0.2048	0.2143
	GGG	0.0718	1.03E-06	0.0962	0.1983	0.1509	0.1871	0.1618	0.1654
	GGT	0.0521	3.99E-04	**0.3268**	0.2590	0.3082	0.2812	0.3015	0.2934
His	CAC	**-0.2282**	**1.10E-55**	**0.5230**	**0.5528**	0.3774	0.4562	0.4316	0.4534
	CAT	0.2416	2.24E-62	0.4770	0.4472	**0.6226**	**0.5438**	**0.5684**	**0.5466**
Ile	ATA	0.3847	6.16E-163	0.0658	0.1715	0.2556	0.2280	0.1916	0.1986
	ATC	**-0.5357**	**1.00E-223**	**0.7076**	**0.6101**	**0.4055**	**0.4772**	**0.5309**	**0.5220**
	ATT	0.3422	3.55E-127	0.2266	0.2185	0.3389	0.2948	0.2776	0.2793
Leu	TTA	0.4527	2.16E-232	0.0324	0.0741	0.1317	0.1209	0.0796	0.0942
	TTG	0.2742	1.67E-80	0.1136	0.1290	0.1641	0.1623	0.1417	0.1459
	CTA	0.2531	1.65E-68	0.0864	0.1046	0.1411	0.1241	0.1305	0.1235
	CTC	**-0.4612**	**2.87E-242**	**0.3035**	**0.2813**	0.1734	**0.2168**	0.2199	0.2267
	CTG	-0.3895	2.64E-167	0.2993	0.2663	0.1917	0.1953	**0.2503**	**0.2339**
	CTT	0.2080	2.28E-46	0.1649	0.1447	**0.1979**	0.1806	0.1780	0.1757
Lys	AAA	0.4662	2.95E-248	0.2195	0.3375	0.4490	0.4707	0.3577	0.3829
	AAG	**-0.4665**	**1.36E-248**	**0.7805**	**0.6625**	**0.5510**	**0.5293**	**0.6423**	**0.6171**
Phe	TTC	**-0.3821**	**1.43E-160**	**0.6961**	**0.6610**	0.4986	**0.5662**	**0.5772**	**0.5841**
	TTT	0.3813	7.54E-160	0.3039	0.3390	**0.5014**	0.4338	0.4228	0.4159
Pro	CCA	0.1854	5.08E-37	**0.3162**	0.2661	**0.4055**	**0.3238**	**0.3471**	**0.3379**
	CCC	**-0.3173**	**1.19E-108**	0.2907	**0.2864**	0.1607	0.2120	0.2215	0.2236

	CCG	-0.0108	4.64E-01	0.1078	0.1889	0.0880	0.1927	0.1019	0.1330
	CCT	0.1568	7.61E-27	0.2853	0.2585	0.3458	0.2715	0.3296	0.3055
Ser	AGC	-0.2299	1.61E-56	0.2111	**0.2121**	0.1497	0.1654	0.1842	0.1796
	AGT	0.2037	1.68E-44	0.1392	0.1558	0.2080	0.1677	0.1900	0.1805
	TCA	0.2309	5.12E-57	0.1460	0.1654	**0.2525**	**0.2077**	**0.2079**	**0.2065**
	TCC	**-0.2789**	**2.19E-83**	**0.2333**	0.1971	0.1258	0.1506	0.1681	0.1645
	TCG	-0.0224	1.27E-01	0.0813	0.1366	0.0585	0.1463	0.0684	0.0940
	TCT	0.1907	3.94E-39	0.1890	0.1330	0.2055	0.1623	0.1815	0.1750
Thr	ACA	0.2837	2.66E-86	0.2151	0.2550	**0.3990**	**0.3125**	0.3235	**0.3171**
	ACC	**-0.4199**	**6.96E-197**	**0.4664**	**0.3608**	0.2339	0.2690	**0.3268**	0.3123
	ACG	-0.0391	7.81E-03	0.1168	0.2267	0.1063	0.2162	0.1308	0.1584
	ACT	0.2770	3.53E-82	0.2016	0.1575	0.2608	0.2023	0.2189	0.2122
Tyr	TAC	**-0.3811**	**1.12E-159**	**0.7296**	**0.6901**	0.4892	**0.5711**	**0.5892**	**0.5955**
	TAT	0.3867	9.07E-165	0.2704	0.3099	**0.5108**	0.4289	0.4108	0.4045
Val	GTA	0.3086	1.48E-102	0.1357	0.1431	0.2329	0.1878	0.1803	0.1832
	GTC	**-0.3935**	**4.42E-171**	**0.4005**	**0.3736**	0.2378	**0.3045**	**0.3047**	**0.3099**
	GTG	-0.0946	1.17E-10	0.2758	0.3089	**0.2695**	0.2685	0.3006	0.2873
	GTT	0.3459	4.56E-130	0.1880	0.1744	0.2598	0.2392	0.2143	0.2196

[a] The most significant negative correlation are highlighted in bold.

[b] The probability a given gene in the group will use the synonymous codon is taken as the relative frequency of the codon in its family for all genes in the group. The highest frequency of a codon family is in bold.

[c] The relative frequency of the synonymous codon for the amino acid for all genes. The highest frequency of a codon family is in bold.
