# Supplementary material for: Genome-Wide Patterns of Codon Bias Are Shaped by Natural Selection in the Purple Sea Urchin, Strongylocentrotus purpuratus
Source: G3 (Bethesda). 2013 Jul 1;3(7):1069–83. doi: 10.1534/g3.113.005769 (PMC3704236; doi:10.1534/g3.113.005769)
Supplement: Supporting Information [file supp_g3.113.005769_TableS4.pdf]

Table S4 The strongest and most significant Spearman correlation coefficients for each of the first three SCUMBLE offsets for a SCUMBLE model with 4 trends.

Group	β_1		β_2		β_3	
0	GC3, 0.8443	***	72h[a], 0.4053	***	CT3, 0.5120	***
1	GC3, 0.8545	***	CT3, 0.1748	***	CT3, 0.4917	***
2	GC3, 0.7724	***	GT3, 0.3189	***	CT3, 0.4735	***
3	GC3, 0.8108	***	CT3, 0.3315	***	CT3, 0.4923	***
4	GC3, 0.7089	***	CT3, 0.3015	***	CT3, 0.5142	***
All	GC3, 0.9961	***	72h, 0.3277	***	CT3, 0.4450	***

*** *P-value*< $1x10^{-10}$
* *P-value*< 0.001
[a] Expression value at 72h after fertilization.
