# Supplementary material for: Genome-Wide Patterns of Codon Bias Are Shaped by Natural Selection in the Purple Sea Urchin, Strongylocentrotus purpuratus
Source: G3 (Bethesda). 2013 Jul 1;3(7):1069–83. doi: 10.1534/g3.113.005769 (PMC3704236; doi:10.1534/g3.113.005769)
Supplement: Supporting Information [file supp_g3.113.005769_TableS5.pdf]

Table S5 Correlations between codon bias (Nc), regional GC content and rates of protein evolution in *Strongylocentrotus purpuratus*.

		Spearman's Correlation Coefficient for each group [a]					
		Group 0	Group 1	Group 2	Group 3	Group 4	All
Codon Bias							
Nc	GC3	-0.5524***	-0.4653***	0.2628***	0.1435***	-0.0202	-0.054*
Nc	GC_{cds}	-0.442***	-0.2217***	0.2394***	0.0896	0.0255	-0.0219
Nc	GC_i[b]	-0.0431	0.0196	0.1365*	0.0365	0.0098	0.0967*
Nc	GC_f	-0.002	-0.0274	0.102*	-0.0314	0.0125	0.0555*
Rate Comparisons[c]							
Nc	*dS*	-0.0237	-0.1361*	-0.0031	-0.0083	-0.019	-0.0519
Nc	*dN*	0.1401	0.1507*	0.0644	0.0955	0.1111	0.1293***
Nc	*dN/dS*	0.1541	0.1967*	0.0557	0.0982	0.1076	0.1407***

* Significance at $P < 0.001$

*** Significance at $P < 10^{-10}$

[a] Number of genes in each group: All (4623), Cluster 0 (396), Cluster 1 (861), Cluster 2 (1154), Cluster 3 (912), Cluster 4 (1300)

[b] Number of genes with introns for each group: All (4389), Cluster 0 (368), Cluster 1 (814), Cluster 2 (1113), Cluster 3 (826), Cluster 4 (1268)

[c] Number of genes with comparative data for each group: All (2954), Cluster 0 (225), Cluster 1 (593), Cluster 2 (744), Cluster 3 (563), Cluster 4 (829)
