# Supplementary material for: Genome-Wide Patterns of Codon Bias Are Shaped by Natural Selection in the Purple Sea Urchin, Strongylocentrotus purpuratus
Source: G3 (Bethesda). 2013 Jul 1;3(7):1069–83. doi: 10.1534/g3.113.005769 (PMC3704236; doi:10.1534/g3.113.005769)
Supplement: Supporting Information [file supp_g3.113.005769_TableS6.pdf]

Table S6. Significantly Over-Enriched Gene Ontology (GO) codes for *S. purpuratus* gene groups.

Group	GO_ID	GO Term	Domain[a]	FDR[b]	*P-value*
C0	GO:0005198	structural molecule activity	F	0	0
C0	GO:0005840	ribosome	C	3.67E-36	5.20E-38
C0	GO:0030529	ribonucleoprotein complex	C	3.67E-36	5.20E-38
C0	GO:0006412	translation	P	2.05E-33	3.86E-35
C0	GO:0032991	macromolecular complex	C	3.14E-32	7.41E-34
C0	GO:0005829	cytosol	C	7.27E-25	2.06E-26
C0	GO:0043228	non-membrane-bounded organelle	C	2.04E-20	7.71E-22
C0	GO:0043232	intracellular non-membrane-bounded organelle	C	2.04E-20	7.71E-22
C0	GO:0003723	RNA binding	F	4.59E-14	1.95E-15
C0	GO:0005737	cytoplasm	C	1.91E-13	8.99E-15
C0	GO:0000166	nucleotide binding	F	7.13E-12	3.70E-13
C0	GO:0030312	external encapsulating structure	C	1.61E-11	9.12E-13
C0	GO:0044444	cytoplasmic part	C	3.40E-11	2.17E-12
C0	GO:0005929	cilium	C	3.40E-11	2.41E-12
C0	GO:0042995	cell projection	C	3.40E-11	2.41E-12
C0	GO:0005811	lipid particle	C	7.86E-11	5.93E-12
C0	GO:0005618	cell wall	C	1.47E-10	1.18E-11
C0	GO:0043234	protein complex	C	1.63E-10	1.38E-11
C0	GO:0009536	plastid	C	1.02E-09	9.13E-11
C0	GO:0005856	cytoskeleton	C	6.87E-09	6.48E-10
C0	GO:0044424	intracellular part	C	1.63E-08	1.61E-09
C0	GO:0043229	intracellular organelle	C	2.64E-08	2.74E-09
C0	GO:0040007	growth	P	2.06E-07	2.23E-08
C0	GO:0009987	cellular process	P	2.47E-07	2.80E-08
C0	GO:0044267	cellular protein metabolic process	P	3.95E-07	4.66E-08
C0	GO:0005622	intracellular	C	5.87E-07	7.20E-08
C0	GO:0019538	protein metabolic process	P	1.28E-06	1.63E-07

C0	GO:0006996	organelle organization	P	1.64E-06	2.16E-07
C0	GO:0010467	gene expression	P	2.59E-06	3.54E-07
C0	GO:0044464	cell part	C	7.47E-06	1.06E-06
C0	GO:0044249	cellular biosynthetic process	P	1.59E-05	2.56E-06
C0	GO:0009059	macromolecule biosynthetic process	P	1.59E-05	2.56E-06
C0	GO:0034645	cellular macromolecule biosynthetic process	P	1.59E-05	2.56E-06
C0	GO:0005515	protein binding	F	1.59E-05	2.62E-06
C0	GO:0043226	organelle	C	1.59E-05	2.62E-06
C0	GO:0016043	cellular component organization	P	2.51E-05	4.63E-06
C0	GO:0003774	motor activity	F	2.51E-05	4.86E-06
C0	GO:0016818	hydrolase activity, acting on acid anhydrides, in phosphorus-containing anhydrides	F	2.51E-05	4.86E-06
C0	GO:0016817	hydrolase activity, acting on acid anhydrides	F	2.51E-05	4.86E-06
C0	GO:0016462	pyrophosphatase activity	F	2.51E-05	4.86E-06
C0	GO:0017111	nucleoside-triphosphatase activity	F	2.51E-05	4.86E-06
C0	GO:0007010	cytoskeleton organization	P	3.74E-05	7.42E-06
C0	GO:0003676	nucleic acid binding	F	9.70E-05	1.97E-05
C0	GO:0009055	electron carrier activity	F	0.00075772	0.000157263
C0	GO:0009790	embryonic development	P	0.000759207	0.000161152
C0	GO:0044422	organelle part	C	0.000948809	0.000210349
C0	GO:0044446	intracellular organelle part	C	0.000948809	0.000210349
C0	GO:0044237	cellular metabolic process	P	0.00152677	0.000345684
C0	GO:0045182	translation regulator activity	F	0.00187651	0.000433721
C0	GO:0030313	cell envelope	C	0.0020718	0.000488631
C0	GO:0007049	cell cycle	P	0.00259108	0.000623326
C0	GO:0065008	regulation of biological quality	P	0.00356715	0.000874962
C0	GO:0005730	nucleolus	C	0.0039766	0.00099415
C0	GO:0006091	generation of precursor metabolites and energy	P	0.00424169	0.00108043
C0	GO:0000003	reproduction	P	0.00441887	0.0011464
C0	GO:0005488	binding	F	0.00627651	0.00165794

C0	GO:0043170	macromolecule metabolic process	P	0.00630195	0.00169439
C0	GO:0005623	cell	C	0.00859926	0.00235263
C0	GO:0044428	nuclear part	C	0.00925341	0.00257524
C0	GO:0016209	antioxidant activity	F	0.0111415	0.00315326
C0	GO:0005509	calcium ion binding	F	0.0138875	0.00399592
C0	GO:0043167	ion binding	F	0.0154194	0.00465491
C0	GO:0043169	cation binding	F	0.0154194	0.00465491
C0	GO:0046872	metal ion binding	F	0.0154194	0.00465491
C0	GO:0005102	receptor binding	F	0.020271	0.00621516
C0	GO:0019725	cellular homeostasis	P	0.0210874	0.00666442
C0	GO:0042592	homeostatic process	P	0.0210874	0.00666442
C0	GO:0031974	membrane-enclosed lumen	C	0.021475	0.00719209
C0	GO:0043233	organelle lumen	C	0.021475	0.00719209
C0	GO:0070013	intracellular organelle lumen	C	0.021475	0.00719209
C0	GO:0031981	nuclear lumen	C	0.021475	0.00719209
C0	GO:0005773	vacuole	C	0.025567	0.00868312
C0	GO:0005764	lysosome	C	0.0260626	0.00909732
C0	GO:0000323	lytic vacuole	C	0.0260626	0.00909732
C0	GO:0009058	biosynthetic process	P	0.0261996	0.00926873
C0	GO:0005634	nucleus	C	0.0275011	0.0098589
C0	GO:0005815	microtubule organizing center	C	0.0289569	0.0107905
C0	GO:0044430	cytoskeletal part	C	0.0289569	0.0107905
C0	GO:0015630	microtubule cytoskeleton	C	0.0289569	0.0107905
C0	GO:0044260	cellular macromolecule metabolic process	P	0.0289666	0.0109308
C0	GO:0008219	cell death	P	0.0320537	0.0122469
C0	GO:0032502	developmental process	P	0.0342324	0.0132408
C0	GO:0030246	carbohydrate binding	F	0.0364777	0.0142814
C0	GO:0016265	death	P	0.03887	0.0154444
C0	GO:0005886	plasma membrane	C	0.03887	0.015768

C0	GO:0016020	membrane	C	0.03887	0.015768
C0	GO:0016049	cell growth	P	0.0447576	0.0190009
C0	GO:0090066	regulation of anatomical structure size	P	0.0447576	0.0190009
C0	GO:0032535	regulation of cellular component size	P	0.0447576	0.0190009
C0	GO:0008361	regulation of cell size	P	0.0447576	0.0190009
C1	GO:0030528	transcription regulator activity	F	3.04E-18	2.13E-20
C1	GO:0006350	transcription	P	3.04E-18	2.86E-20
C1	GO:0090304	nucleic acid metabolic process	P	8.06E-14	2.66E-15
C1	GO:0003700	transcription factor activity	F	3.64E-12	1.37E-13
C1	GO:0006139	nucleobase, nucleoside, nucleotide and nucleic acid metabolic process	P	1.48E-10	7.66E-12
C1	GO:0006807	nitrogen compound metabolic process	P	1.48E-10	7.66E-12
C1	GO:0034641	cellular nitrogen compound metabolic process	P	1.48E-10	7.66E-12
C1	GO:0008283	cell proliferation	P	5.31E-07	3.26E-08
C1	GO:0023052	signaling	P	1.44E-06	1.06E-07
C1	GO:0009653	anatomical structure morphogenesis	P	2.40E-06	2.04E-07
C1	GO:0048856	anatomical structure development	P	2.40E-06	2.04E-07
C1	GO:0007165	signal transduction	P	5.54E-06	5.75E-07
C1	GO:0023046	signaling process	P	5.54E-06	5.75E-07
C1	GO:0050794	regulation of cellular process	P	5.54E-06	5.75E-07
C1	GO:0023060	signal transmission	P	5.54E-06	5.75E-07
C1	GO:0030154	cell differentiation	P	5.63E-06	6.37E-07
C1	GO:0048869	cellular developmental process	P	5.63E-06	6.37E-07
C1	GO:0003677	DNA binding	F	2.11E-05	2.49E-06
C1	GO:0005215	transporter activity	F	5.54E-05	6.80E-06
C1	GO:0007267	cell-cell signaling	P	0.000245924	3.25E-05
C1	GO:0007610	behavior	P	0.000478496	6.55E-05
C1	GO:0051179	localization	P	0.000484876	7.45E-05
C1	GO:0006810	transport	P	0.000484876	7.45E-05
C1	GO:0051234	establishment of localization	P	0.000484876	7.45E-05

C1	GO:0032501	multicellular organismal process	P	0.000484876	7.55E-05
C1	GO:0007275	multicellular organismal development	P	0.00049517	7.94E-05
C1	GO:0050789	regulation of biological process	P	0.00277685	0.000484638
C1	GO:0006811	ion transport	P	0.00373494	0.000687088
C1	GO:0022857	transmembrane transporter activity	F	0.00439264	0.0008288
C1	GO:0065007	biological regulation	P	0.00448243	0.000866886
C1	GO:0043227	membrane-bounded organelle	C	0.00505752	0.00112408
C1	GO:0043231	intracellular membrane-bounded organelle	C	0.00505752	0.00112408
C1	GO:0005216	ion channel activity	F	0.00505752	0.00119281
C1	GO:0022892	substrate-specific transporter activity	F	0.00505752	0.00119281
C1	GO:0022803	passive transmembrane transporter activity	F	0.00505752	0.00119281
C1	GO:0022891	substrate-specific transmembrane transporter activity	F	0.00505752	0.00119281
C1	GO:0015267	channel activity	F	0.00505752	0.00119281
C1	GO:0022838	substrate-specific channel activity	F	0.00505752	0.00119281
C1	GO:0015075	ion transmembrane transporter activity	F	0.00505752	0.00119281
C1	GO:0007154	cell communication	P	0.0073416	0.00176614
C1	GO:0003682	chromatin binding	F	0.00852481	0.00209099
C1	GO:0006464	protein modification process	P	0.0140444	0.00384233
C1	GO:0043412	macromolecule modification	P	0.0140444	0.00384233
C1	GO:0003779	actin binding	F	0.0234774	0.00686602
C1	GO:0008289	lipid binding	F	0.0321267	0.00998075
C1	GO:0030234	enzyme regulator activity	F	0.0411592	0.0141727
C2	GO:0005739	mitochondrion	C	0.00106027	2.50E-05
C2	GO:0003824	catalytic activity	F	0.00159266	6.14E-05
C2	GO:0015031	protein transport	P	0.00159266	8.70E-05
C2	GO:0033036	macromolecule localization	P	0.00159266	8.70E-05
C2	GO:0008104	protein localization	P	0.00159266	8.70E-05
C2	GO:0045184	establishment of protein localization	P	0.00159266	8.70E-05
C2	GO:0005794	Golgi apparatus	C	0.00159266	8.79E-05

C2	GO:0006259	DNA metabolic process	P	0.00280385	0.000198386
C2	GO:0016788	hydrolase activity, acting on ester bonds	F	0.026659	0.00238925
C3	GO:0009719	response to endogenous stimulus	P	0.000191016	4.51E-06
C3	GO:0005975	carbohydrate metabolic process	P	0.00102028	3.85E-05
C3	GO:0044421	extracellular region part	C	0.00651626	0.000430319
C3	GO:0005615	extracellular space	C	0.00995884	0.000845561
C3	GO:0016740	transferase activity	F	0.0182658	0.00172319
C3	GO:0005768	endosome	C	0.0316677	0.00388378
C3	GO:0044238	primary metabolic process	P	0.0337436	0.00429753
C4	GO:0008152	metabolic process	P	7.88E-06	1.08E-06
C4	GO:0004672	protein kinase activity	F	8.30E-05	1.46E-05
C4	GO:0016773	phosphotransferase activity, alcohol group as acceptor	F	8.30E-05	1.46E-05
C4	GO:0016772	transferase activity, transferring phosphorus-containing groups	F	8.30E-05	1.53E-05
C4	GO:0016301	kinase activity	F	8.30E-05	1.53E-05
C4	GO:0009056	catabolic process	P	0.000764796	0.000151516
C4	GO:0016787	hydrolase activity	F	0.00104996	0.000232775
C4	GO:0016032	viral reproduction	P	0.0254601	0.00744587

[a]GO domain: cellular component (‘C’), biological process (‘B’) and molecular function (‘F’)
[b] False Discovery Rate
